# Mechanical stimulation simulating osteopathic pressing manipulation inhibits nociceptive hypersensitivity and synovial inflammation in knee osteoarthritis rats by modulating trafficking dynamics of transient receptor potentials via complexin2

**DOI:** 10.1186/s13018-025-06414-7

**Published:** 2025-11-05

**Authors:** Li Zhang, Hua Zhang, Ping Li, Guangjuan Ke, Song Gao, Liuxin Qu

**Affiliations:** 1 Department of Traditional Chinese Orthopedics and Traumatology, Zhongda Hospital, School of Medicine, Southeast University, Nanjing, China; 2 Institute of Neuropsychiatry, Zhongda Hospital, School of Medicine, Southeast University, Nanjing, China; 3 Nanjing, No.87 Dingjiaqiao, Gulou District Jiangsu China

**Keywords:** Knee osteoarthritis, Nociceptive hypersensitivity, Synovial inflammation, SNAP25 VAMP2, Complexin2

## Abstract

Nociceptive hypersensitivity and synovial inflammation promote the progression of knee osteoarthritis (KOA) and joint pain, yet effective therapeutic strategies remain limited. This study investigated the mechanism of osteopathic pressing manipulation (OPM), a traditional Chinese spinal orthopedic technique, in alleviating KOA pain and inflammation. Using a rat KOA model, we demonstrated that moderate-intensity OPM (MOPM) significantly improved mechanical withdrawal threshold and reduced synovial inflammation markers IL-6, TNF-α and nociceptors TRPV1, TRPA1. RNA sequencing of dorsal root ganglion (DRG) tissues revealed that MOPM reversed KOA-induced upregulation of complexin 2 (CPLX2), a key modulator of SNARE complex-mediated cellular membrane trafficking. Mechanistically, MOPM suppressed CPLX2-dependent vesicle transport, thereby reducing membrane localization of TRP channels and secretion of pain mediators NGF, CGRP. In vitro co-culture experiments using DRG neurons and fibroblast-like synoviocytes confirmed that mechanical stress mimicking OPM attenuated trafficking dynamics-related protein expression VAMP2, SNAP25, and syntaxin1 via CPLX2 downregulation. Critically, CPLX2 siRNA synergized with MOPM to enhance anti-nociceptive and anti-inflammatory effects. These findings identify OPM as a non-pharmacological intervention capable of modulating CPLX2-mediated neuronal trafficking dynamics of TRPV1 and TRPA1, thereby alleviating pain and inflammation associated with KOA.

**Supplementary Information:**

The online version contains supplementary material available at 10.1186/s13018-025-06414-7.

## Introduction

Knee osteoarthritis (KOA) affects nearly 7% of the population worldwide, and the incidence is increasing year by year [1, 2]. Intractable chronic pain is the most prominent clinical symptom in KOA patients, and also an important factor for the loss of joint function in the end-stage, indicating a huge medical burden [3]. In recent authoritative guidelines, KOA pain is clearly defined as a mixed pain combining nociceptive and neuropathic, thus pain control and symptom relief are recommended as the primary goal of KOA treatment [4, 5]. Notably, KOA pain has definite nociceptive hypersensitivity, which is associated with constant stimulation of synovial inflammation, overexpression of nociceptors, and abnormal activity of sensory nerve fibers [6].

Various ion channels on the surface of the neuronal cell membrane may act as nociceptors of KOA to sense different stimuli and transmit pain signals, and the transient receptor potentials (TRPs) channel superfamily is the most thoroughly studied [7, 8]. These organic cation channels are activated in terms of different stimulus conditions, such as temperature, osmotic pressure, or chemicals, then mediate the intracellular flow of Ca^2+^ to initiate intracellular signaling [7–9]. Major pro-inflammatory factors in synovial inflammation, such as interleukin-6 (IL-6) and tumor necrosis factor-α (TNF-α), have been reported to up-regulate the expression levels of TRPV1 and TRPA1, and aggravate the pain behavior of KOA animals [10, 11]. In addition, localized injections of the side-directed TRPV1 agonist Capsaicin and Resiniferatoxin are undergoing clinical trials in patients with KOA and bone cancer pain [12]. However, little is known about how the TRPs protein inserts into the cell membrane after transcriptional synthesis and plays a role in KOA pain.

Existing studies support the co-localization of TRPV1 and TRPA1 in neuronal membrane trafficking, both of which are accomplished by vesicle transport mediated by the soluble N-ethylmaleimide-sensitive factor attachment protein receptor (SNARE) complex [13, 14]. The SNARE complex consists of a parallel four-helix bundle and brings the vesicle and plasma membranes together. Typically, the SNARE complex involved in the above process consists of syntaxin1, synaptosomal-associated protein of 25 kDa (SNAP25), and synaptobrevin-2 (also called vesicle-associated membrane protein 2, VAMP2) [15]. Among them, syntaxin-1 is a protein that is specifically distributed on the synapses or dendritic plasma membranes of neurons, and SNAP25 is attached to the plasma membrane by palmitoylation, both of which are the key proteins for cell plasma membrane fusion and called t-SNARE; while VAMP2 is often referred to as a v-SNARE because of its vesicle localization [16–18]. In addition to TRPV1 or TRPA1, similar vesicle transport mechanisms occur in the process of neuronal secretion of pain mediators such as calcitonin gene related peptide (CGRP), nerve growth factor (NGF), and therefore, intervention strategies targeting these pathways have been shown to be effective for nociceptive hypersensitivity caused by nerve damage [19].

In KOA, the TRPs channel is sensitized by persistent noxious stimulation, and this stimulation-mediated electrical signal is transmitted through nerve fibers, eventually processed in the dorsal root ganglion (DRG) tissue [20]. Nerve fibers originating from 2–4 lumbar DRG innervate the area around the knee joint, so the DRG of L2-4 eventually transmits pain signals along the “dorsal horn of the spinal cord—cerebral cortex” via ganglion substitution, causing pain perception [21]. Therefore, drug delivery targeting DRG neurons support a future therapeutic approach in the pain management of KOA [22, 23].

In addition to medication, physical therapy, including acupuncture, massage, and manipulative therapies, is often effective in treating KOA pain, and the location of the operation is often not limited to the local knee joint, but often from the waist. For example, the distal point selection of acupuncture treatment of KOA is mostly located on the waist [24]. Our previous work demonstrated the effectiveness of traditional Chinese spinal orthopedic manipulation (TCSOM) in the treatment of KOA pain and chondromalacia patellae [25]. This traditional Chinese spinal orthopedic manipulation mainly includes osteopathic pressing manipulation (OPM) with thumb and obliquely pulling manipulation performed at L2 to L4 according to patient’s symptoms, signs and examinations [25, 26]. Within this context, we speculated that the mechanical stimulation induced by osteopathic compression manipulation may cause some changes in the transcription level of DRG genes, thereby inhibiting the nociceptive hypersensitivity and synovial inflammation of KOA. In this study, we combined RNA sequencing (RNA-seq) and molecular biology techniques to investigate the mechanism of Osteopathic pressing manipulation in the treatment of KOA nociceptive hypersensitivity and synovial inflammation.

## Material and methods

### Animals and modeling

SD rats weighed 180 to 220 g were purchased from Charles River Laboratories and fed in a specific pathogen-free, laminar-flow housing apparatus. Breeding was under 25 ± 2 °C temperature, 55 ± 5% humidity, and 12 h light/dark regimen. All animal protocols were approved by the Animal Care and Use Committee of the Southeast University (No: 20240220015).

According to our previous study [6], the KOA model (bilateral knee joints) was established by transecting the anterior cruciate ligament (ACLT). The Sham group underwent a sham procedure, in which the joint capsule was opened without ACLT, followed by layered suturing. Fourteen days after surgery, the KOA model was established successfully.

### In vivo experiment design and OPM protocol

In order to apply OPM to rats, we designed OPM device for small animals, which is composed of sleeve, spring, connecting rod and rubber cap. When OPM is routinely performed on adult patients with KOA, the downward pressure of the practitioner is 100 N [25, 26], and the indent of the thumb abdomen is 2 cm*1 cm oval, so the pressure is 100 N/1.57 cm^2^. Based on the muscle tissue of human body and small animal bear the same pressure, and the contact area between the device rubber cap and the experimental animal is 0.1256 cm^2^, to simulate clinical pressure under 100 N needs to apply 8 N pressure to the small animal. The spring in the device can provide 2 N forces per 1 mm compression, so when the spring is compressed by 4 mm, the clinical OPM can be simulated. We assumed that the compression of 4 mm is a medium-intensity OPM (MOPM), then the compression of 2 mm is a low-intensity OPM (LOPM), and the compression of 16 mm is a high- intensity OPM (HOPM).

Subsequently, 40 rats were randomly divided into 5 groups, namely, Sham, KOA, KOA + LOPM, KOA + MOPM, and KOA + HOPM. Except the Sham group, all other groups underwent ACTL surgery to construct KOA model. OPM was intervened to rats with the device designed above. According to the surface landmark method, the rat’s iliac crest (the highest point of the pelvis) can be palpated. When the thumb is placed at the highest point of the iliac crest, the tip of the thumb points to the intervertebral disc located between the fourth and fifth lumbar vertebrae. Our procedure was performed collaboratively by two experimenters: one was responsible for anatomical localization, while the other operated the OPM. The rats were placed in a quiet environment. After the rats stopped exploring, the rubber cap of the device was placed vertically next to the spine process of the L3-5 lumbar vertebrae and the device was pressed vertically according to the different intensity of the corresponding groups, for 2 s in each place, cycling for 2 min, once every other day, for a total of 28 days.

In another set, 32 rats were randomly divided into Sham, KOA, KOA + MOPM, KOA + MOPM + CPLX2 siRNA. Each group was given a corresponding intervention according to their names, in particular, KOA + MOPM + CPLX2 siRNA group received KOA modeling, MOPM, commercial lentivirus mediated CPLX2 siRNA 20 μL (1 × 10^8^ TU/mL, RiboBio, China) for intraarticular injection, once a week for two weeks. Meanwhile, the other groups received intraarticular injections of empty lentivirus vectors carrying non-targeting siRNA sequences, provided by the commercial lentivirus kit, at the same frequency, to serve as controls.

### Cell culture

Primary rat fibroblast-like synoviocytes (FLSs) were extracted as our method described previously [11]. Briefly, synovial tissues were harvested and minced into pieces of 2-3mm^2^, digested with 0.2% collagenase type I at 37 °C for 1 h. Digestion were terminated by the addition of 10% fetal bovine serum (FBS, Gibco, USA). FLSs were cultured in complete medium, namely DMEM/F12 medium supplemented with 10% FBS and 1% penicillin and streptomycin, incubated under standard conditions (37 °C, 5% CO_2_), used for up to five generations in all vitro experiments.

Primary rat DRG neurons were extracted as the method described previously [21]. Briefly, rat spinal column was quickly separated after execution, and the pyramidal body was cut from the foramen along the median line, divided into two halves, and placed in MEM medium containing 2% serum. After removing the excess tissue, DRG tissue of the corresponding segment was obtained from the lateral recess, and placed in mixed digestive enzymes containing type I collagenase and disperse enzyme 5 mg/mL, and digested in water bath for 60 min. Shake it upside-down every 10 min, then completely suspend the medium, centrifuge at 1000 rpm for 8.5 min, and finally inoculated in culture dishes coated with Fibronectin solution.

### In vitro experiment design and FX-6000 T stress loading system

DRG neurons were rapidly isolated and inoculated on the special elastic 6-well plate of FX-6000 T cell stress loading system. This system is capable of applying both axial and circumferential stress to two-dimensional and three-dimensional cell and tissue cultures. In our study, this system was used in conjunction with the Flex Flow parallel plate, allowing the simultaneous application of fluid shear stress while stretching the cells. After 8 h, complete medium containing 1% 5-fluorouracil was used for continuous incubation to inhibit the proliferation of satellite glial cells. FLSs pre-inoculated in Transwell chambers were mounted on the 6-well plates to establish a co-culture system for DRG neuron cells. Subsequently, except Sham group, the other groups were treated with LPS 5 μg/mL for 8 h to simulate the inflammatory environment of KOA. The in vitro intervention of OPM was simulated by the FX-6000 T cell stress loading system. The parameter setting [27] was sine wave, 1 HZ, for 6 h, and the amplitude was set to 2%, 4%, and 8% according to the LOPM, MOPM, and HOPM, respectively.

### RNA-seq

DRG tissues were collected from three groups: Sham, KOA, and KOA + MOPM. Total RNA extraction was accomplished using the EZ-press RNA Purification Kit, following the manufacturer’s instructions. RNA concentration and purity were measured via a NanoDrop 2000 spectrophotometer (Thermo Scientific, USA), while RNA integrity was determined on an Agilent 2100 Bioanalyzer (Agilent Technologies, USA). Only RNA samples with an Integrity Number greater than 7 were selected for subsequent steps. The mRNA library preparation was carried out using the TruSeq Stranded mRNA LT Sample Prep Kit (Illumina, USA), in compliance with the manufacturer’s guidelines [28]. Libraries were then sequenced using the Illumina HiSeq TM3000 platform to obtain 125 or 150 bp paired-end reads.

Sequencing data underwent mapping, quantification, and filtering before differential gene expression analysis. Identification of differentially expressed genes (DEGs) was performed with Limma, edgeR, and DESeq2 packages, considering those with |fold change|≥ 1.5 and *P* < 0.05 as significant [29]. Gene Ontology (GO) enrichment analyses were conducted, and visualizations generated using the OmicShare online suite. Additionally, gene set enrichment analysis (GSEA) was applied with GSEA software provided by the Broad Institute.

### Immunofluorescence

Fixed tissue sections or cell slides were heated at 95 °C for 15 min and treated with 3% H_2_O_2_ for 30 min, then brightened with 0.5% Triton X-100/PBS for 30 min, blocked with 5% BSA for 1 h at room temperature. Sections were incubated with primary antibodies overnight at 4 °C, and incubated with corresponding fluorescent secondary antibody. The nucleus was stained DAPI for 10 min at room temperature. Images were captured using a laser confocal microscope (Zeiss LSM710, Germany) and saved.

### Histological analysis

Synovium from the surface of the infrapatellar fat pad were collected and embedded in paraffin, sectioned 5 μm thickness, stained with HE, Masson and Sirius red, according to the instructions of the stain kit, respectively. Sections were observed under a Leica DMI3000B microscope, with the bright field. The degree of synovial inflammation in HE sections were evaluated according to Krenn synovitis Score [30].

### Enzyme linked immunosorbent assay (ELISA)

The content of NGF, CGRP, IL-6 and TNF-α in serum or in the culture media were determined using a commercially available rat ELISA kit (Beyotime Biotechnology, China) according to manufacturer’s instructions.

### Quantitative real-time polymerase chain reaction (qPCR)

Total RNA from tissues or cells was extracted by using TRIzol reagent (Invitrogen, USA) according to the manufacturer’s instructions, synthesized to cDNA by reverse transcription (cDNA synthesis kit, Takara, Japan). Primer was designed and synthesized by Shanghai Biotechnology Service Company in accordance with Gene sequence in GenBank Gene sequence design, together with Oligo v6.6 (Sequences as Table 1). Real-time PCR was performed using Premix Ex Taq SYBR-Green PCR (Takara Biotechnology, Japan) according to the manufacturer’s instructions on an ABI PRISM 7500 (Applied Biosystems, USA). The mRNA level of individual genes was normalized to GAPDH and calculated by the 2^−ΔΔCT^data analysis method.

**Table 1:** Nucleotide sequences of primers used for Real-time PCR amplification

Target gene	Forward primer	Reverse primer
IL-6 (NM012589.2)	CCACTTCACAAGTCGGAGG	CAGAAGACCAGAGCAGATTTTC
TNF-α (NM012675.3)	AACACAGACTGTTCCCTGAG	TTCTCTCAATGACCCGTAGG
TRPA1 (NM207608.2)	GAGAATGGGAACACAGCTTTG	TTAACGATGTCAGTGGCTCC
TRPV1 (NM031982.1)	TGACAGCGAGTTCAAAGACC	CTGGCATTGACAAACTGCTT
syntaxin1 (AB467282.1)	ATGACATGATGTGCGTGTGTG	CCAAAGCTTACCAGAAGAGC
SNAP25 (BC087699.1)	TATGGGACTTGCTGGAGGAT	AGAAAACCTGGTGTGTGTGG
VAMP2 (BC074003.1)	AGCAAGCTATTTCCCTGTTGG	ATATTGGGGGTCTAAAACACAAGC
GAPDH (NM017008.4)	AACAGCAACTCCCATTCTTCC	GTTTGGGATGGAATTGTGAGG

#### Western blotting (WB)

Total proteins were obtained by adding RIPA lysate to tissues or cells, TRPs proteins on the surface of DRG neurons were extracted by ProteoExtract® Native Membrane Protein Extraction Kit (Merck-Millipore, China). Protein concentration was measured in accordance with the instructions for the preparation of the BSA standard curve. Then, electrophoresis, membrane transfer, BSA closed, add the corresponding primary antibody (1:1000), incubated with secondary antibody, and exposed.

#### statistical analysis

All experiments were performed independently at least thrice, and data were presented as mean ± standard deviation. Statistical analysis was performed using GraphPad Prism 8.0 Software. The Shapiro–Wilk test was employed to assess the normality of the data, while Levene’s test was used to evaluate the homogeneity of variances. Group comparisons were assessed with Student’s t-test or one-way ANOVA for comparison of multiple columns. A value of *P* < 0.05(two-tailed) was considered as statistically significant.

## Results

### Mechanical stimulation simulating osteopathic pressing manipulation alleviates KOA nociceptive hypersensitivity and synovial inflammation in vivo

**Fig. 1:**
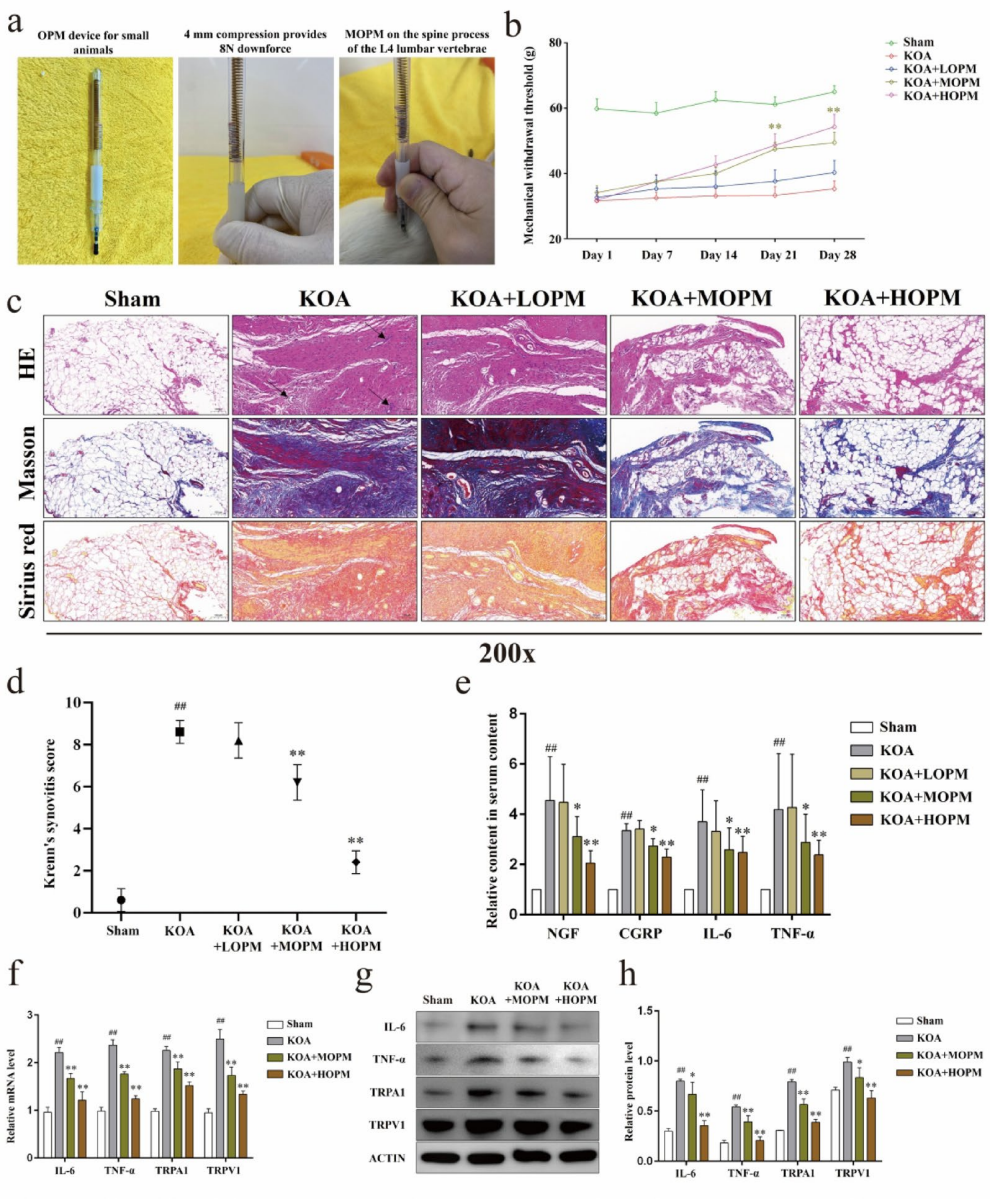
Mechanical stimulation simulating osteopathic pressing manipulation alleviates KOA nociceptive hypersensitivity and synovial inflammation in vivo. *Notes*: **a**. OPM device for small animals and MOPM diagram of rats. **b**. MWT values of rats in each group at different time points, ^**^P < 0.01 vs. KOA group. **c**. Representative HE, Masson and Sirius red staining of rats’ synovial tissue in each group, the black arrows indicate infiltrating inflammatory cells. **d**. Krenn’s synovitis score of HE staining. **e**. The content of serum NGF, CGRP, IL-6, and TNF-α, ^##^*P* < 0.01 vs. Sham, ^*^P < 0.05, ^**^P < 0.01 vs. KOA group. **f**. Relative gene expression of IL-6, TNF-α, TRPA1 and TRPV1 in synovial tissues, ^##^*P* < 0.01 vs. Sham, ^**^P < 0.01 vs. KOA. **g**. Representative protein bands of IL-6, TNF-α, TRPA1 and TRPV1 in synovial tissue. **h**. Relative protein level of IL-6, TNF-α, TRPA1 and TRPV1, ^##^*P* < 0.01 vs. Sham, ^*^P < 0.05, ^**^P < 0.01 vs. KOA

Firstly, we designed a small animal OPM device (Fig. 1a), in which the spring compression of 1 mm can produce 2N downforce, in order to simulate the operation of clinical OPM. Accordingly, we design different OPM intensity groups according to the compression scale of the spring. We measured the mechanical withdrawal threshold (MWT, Fig. 1b) of each group of weekly during a 28-day OPM intervention course. MWT in KOA group were significantly lower than Sham, while moderate-intensity OPM intervention significantly upregulated MWT in KOA rats from Day21 onwards (*P* < *0.01*). The pathological staining (Fig. 1c) showed that compared with the Sham, the synovial tissue of rats in KOA group showed more inflammatory cell infiltration, disordered lining layer cells, and collagen deposition, the above pathological changes were improved in KOA + MOPM and KOA + HOPM groups compared with KOA. Consistently, Krenn’s synovitis score of synovial tissue HE staining (Fig. 1d) in KOA group were higher than those in Sham (*P* < *0.01*), while KOA + MOPM and KOA + HOMP were lower than those in KOA (*P* < *0.01*). Further, we detected the levels of pain mediators NGF, CGRP and proinflammatory factors IL-6, TNF-α in rat serum (Fig. 1e), content of those factors in KOA group was higher than in the Sham (*P* < *0.01*), and medium intensity of OPM could significantly reduce their serum content (*P* < *0.05*). It is worth noting that in the above studies, LOPM didn’t seem to work, so we determined the most basic effective intensity of OPM action on rats and conducted the follow-up in vivo studies. We detected the gene and protein expression of IL-6, TNF-α, and nociceptor TRPA1, TRPV1, in synovial tissues of each group (Fig. 1f–h). Both MOPM and HOPM significantly reversed the up-regulation of the above substances in the KOA model (*P* < *0.05*).

### Mechanical stimulation simulating osteopathic pressing manipulation alleviates nociceptive hypersensitivity and synovial inflammation in vitro

**Fig. 2:**
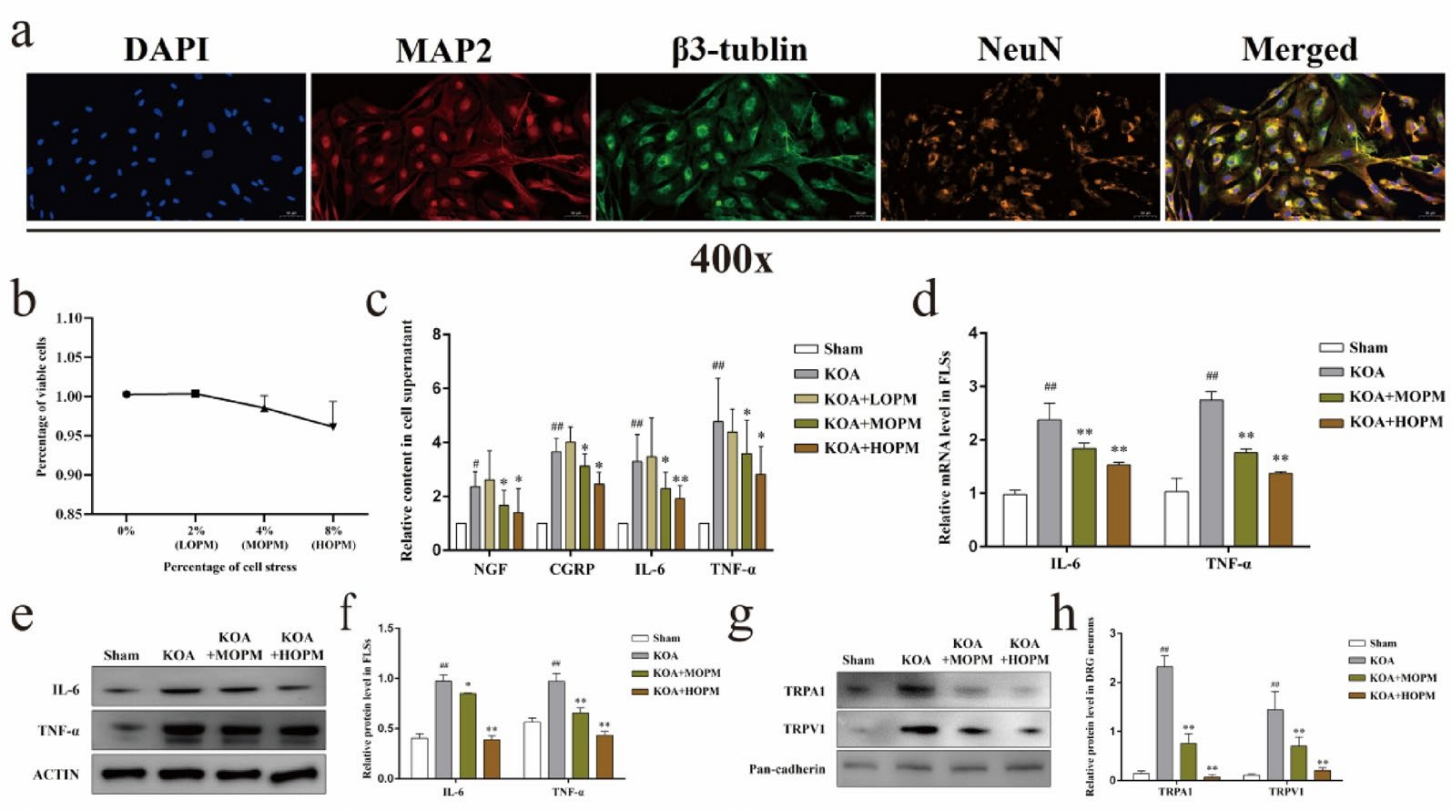
Mechanical stimulation simulating osteopathic alleviates nociceptive hypersensitivity and synovial inflammation in vitro. *Notes*: **a**. Identification of DRG neurons. **b**. Percent of viable DRG neuron cells under different FX-6000 T amplitudes. **c**. The contents of NGF, CGRP, IL-6, and TNF-α in the supernatant of cocultured cells, ^#^*P* < 0.05, ^##^*P* < 0.01 vs. Sham, ^*^P < 0.05, ^**^P < 0.01 vs. KOA group. **d**. Relative gene level of IL-6 and TNF-α in FLSs, ^##^*P* < 0.01 vs. Sham, ^**^P < 0.01 vs. KOA. **e**. Representative protein bands of IL-6 and TNF-α in FLSs. **f**. Relative protein level of IL-6 and TNF-α in FLSs, ^##^*P* < 0.01 vs. Sham, ^*^P < 0.05, ^**^P < 0.01 vs. KOA. **g**. Representative protein bands of TRPA1, TRPV1 in the cell membrane of DRG neurons. **h**. Relative protein expression of TRPA1, TRPV1 in the cell membrane of DRG neurons, ^##^*P* < 0.01 vs. Sham, ^**^P < 0.01 vs. KOA

Next, we isolated and identified rat DRG neurons (Fig. 2a), labeling DAPI as nucleus, neurons as neuronal specific antigen NeuN, dendritic skeleton as microtubule-associated protein 2 (MAP-2), and axonal skeleton as β3-tubulin. We also tested the effects of FX-6000 T cell stress loading system on the survival activity of DRG neurons when used to simulate OPM in vitro, the setting amplitudes of 2%, 4%, or 8% did not change cell survival rates (Fig. 2b). Subsequently, Transwell system was used to establish the cells co-culture between OPM neurons and LPS-treated FLS. In consistent with the results of in vivo, contents of NGF, CGRP, IL-6, and TNF-α in the supernatant of cocultured cells (Fig. 2c) simulated KOA were significantly higher than those in Sham group (*P* < *0.05*), and the above factors were lower in KOA + MOPM and KOA + HOMP groups compared with KOA group (*P* < *0.05*), but not in KOA + LOPM (*P* > *0.05*). In addition, we detected both gene and protein expression of IL-6, TNF-α in FLSs (Fig. 2d–f), and protein expression of TRPA1, TRPV1 in the cell membrane of DRG neurons using a nuclear plasmic separation kit (Fig. 2g, h), respectively. The expression levels of pro-inflammatory factors IL-6 and TNF-α in FLSs were significantly up-regulated in KOA model compared with Sham (*P* < *0.01*), and MOPM could down-regulate their expressions in KOA (*P* < *0.05*). Similarly, relative protein level of nociceptor TRPA1, TRPV1 in DRG neurons were significantly up-regulated in KOA model compared with Sham (*P* < *0.01*), and down-regulate in KOA + MOPM compared with KOA (*P* < *0.05*).

### RNA-seq reveals the intervention effect of OPM on KOA may be related to the complexin2-mediated DRG cellular membrane trafficking

**Fig. 3:**
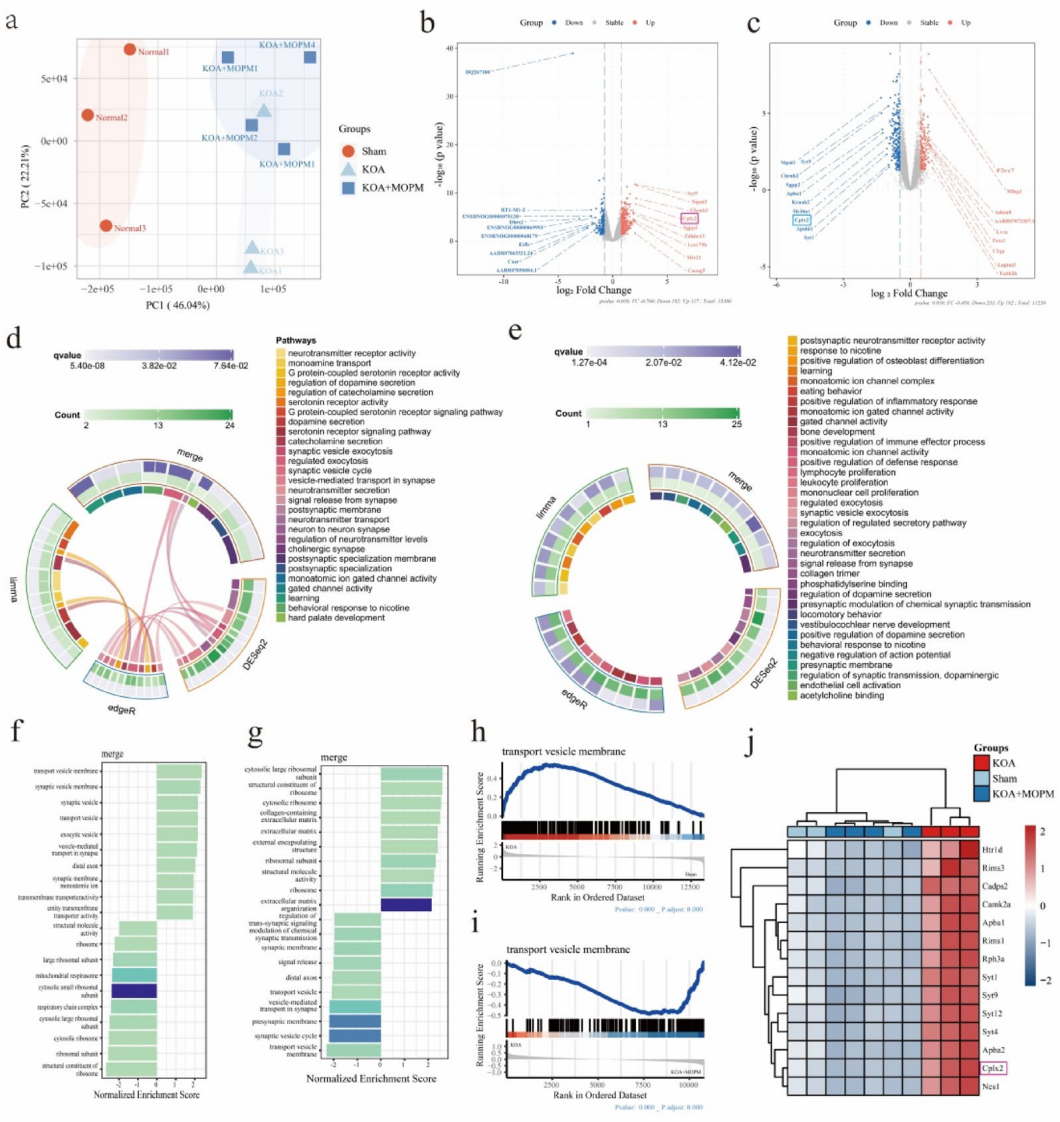
RNA-seq reveals the intervention effect of OPM on KOA may be related to the complexin2-mediated DRG cellular membrane trafficking. *Notes*: **a**. Individual-PCA plot of gene expression in Sham (circle), KOA (square), and KOA + MOPM (triangle) samples revealed strong clustering of samples by phenotype. **b**. Volcano plot representation of DEGs analysis in KOA vs. Sham group. **c**. Volcano plot representation of DEGs analysis in KOA + HOPM vs. KOA. **d**. String plot revealed GO analysis Enrich circle conducted by DEGs between KOA vs. Sham. **e**. String plot revealed GO analysis Enrich circle conducted by DEGs between KOA + HOPM vs. KOA. **f**. KEGG analysis conducted by DEGs between KOA vs. Sham. **g**. KEGG analysis conducted by DEGs between KOA + HOPM vs. KOA. **h**. GSEA analysis of DEGs enriched in transport vesicle membrane pathways between Sham and KOA. **i**. GSEA analysis of DEGs enriched in transport vesicle membrane pathways between KOA + HOPM vs. KOA. **j**. Heat map of DEGs enriched in transport vesicle membrane pathways between the three groups Up

To better understand which genes are involved in the intervention of OPM on KOA, we conducted RNA sequencing of DRG tissues obtained from the Sham, KOA and MOPM group, on the basis of confirming that MOPM can effectively alleviates nociceptive hypersensitivity and synovial inflammation of KOA in vivo and in vitro. The principal components analysis (PCA, Fig. 3a) revealed strong clustering of samples by phenotype. Compared with the Sham, DRG tissue of KOA group identified 317 up-regulated genes and 192 down-regulated genes (Fig. 3b), while KOA + MOPM group identified 192 up-regulated genes and 233 down-regulated genes compared with the KOA (Fig. 3c). Remarkably, upregulation of the gene complexin2 (CPLX) in KOA was significantly reversed by MOPM (Fig. 3b, c). Our Go enrichment analysis string plot revealed pathways related to neurotransmitter signaling and synaptic function pathways (Fig. 3d, e). Use three differential analysis methods, limma, DESeq2, and edgeR, the most significant enrichment pathways are mainly related to the synaptic function: including synaptic vesicle exocytosis, regulation of exocytosis, and vesicle mediated transport; neurotransmitter system: including neurotransmitter signaling pathways such as dopamine secretion, serotonin receptor signaling, and catecholamine secretion pathways; structural components: involving postsynaptic membrane and specialization. Besides, KEGG analysis showed that DEGs significantly up-regulated in the KOA group (vs. Sham group) and significantly down-regulated in the KOA + MOPM group (vs. KOA group) were significantly enriched in transport vesicle membrane and synaptic vesicle membrane pathways (Fig. 3f, g). GSEA plots evaluating the changes of transport vesicle membrane related genes, and genes in this category were upregulated in KOA group (Fig. 3h) and downregulated in KOA + MOPM group (Fig. 3i). In addition, heat map (Fig. 3j) showed DEGs enriched in transport vesicle membrane pathways between the three groups, which were consistent with the results of volcano map, the gene cplx2 in KOA was significantly reversed by MOPM.

### Membrane trafficking dynamics of DRG neurons in KOA is regulated by CPLX2 in vivo

**Fig. 4:**
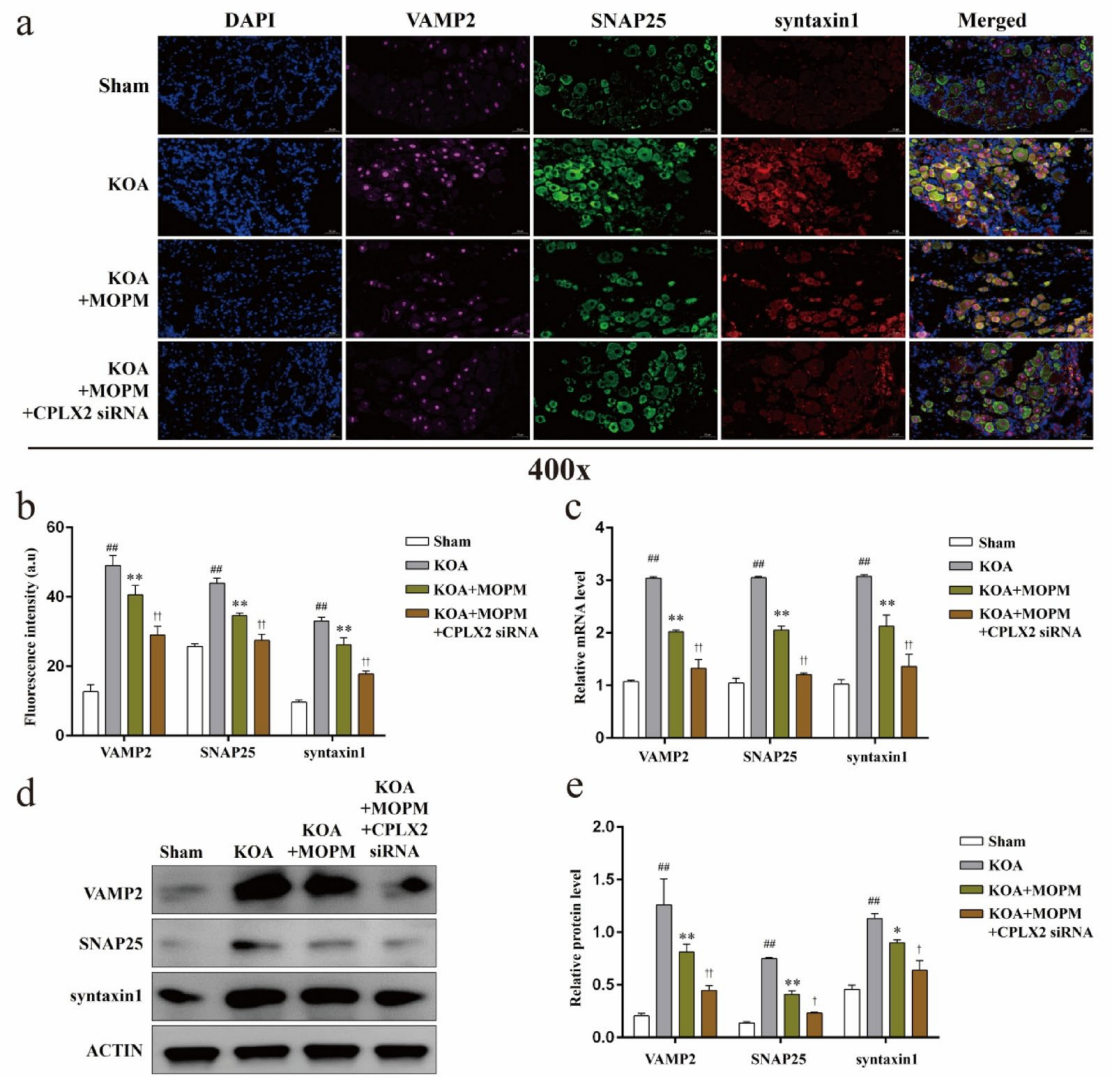
Membrane trafficking dynamics of DRG neurons in KOA is regulated by CPLX2 in vivo. *Notes*: **a**. Representative immunofluorescence of VAMP2 (purple), SNAP25 (green), and syntaxin1 (red) in DRG tissues. **b**. Fluorescence intensity statistics of VAMP2, SNAP25, and syntaxin1 in each group, ^##^*P* < 0.01 vs. Sham, ^**^P < 0.01 vs. KOA, ^††^*P* < 0.01 vs. KOA + MOPM. **c**. Relative gene level of VAMP2, SNAP25, and syntaxin1 in DRG tissues, ^##^*P* < 0.01 vs. Sham, ^**^P < 0.01 vs. KOA, ^††^*P* < 0.01 vs. KOA + MOPM. **d**. Representative protein bands of VAMP2, SNAP25, and syntaxin1 in DRG tissues. **f**. Relative protein level of VAMP2, SNAP25, and syntaxin1 in DRG tissues, ^##^*P* < 0.01 vs. Sham, ^*^P < 0.05, ^**^P < 0.01 vs. KOA, ^†^*P* < 0.05, ^††^*P* < 0.01 vs. KOA + MOPM

CPLX2 is an important member of the complexin family, interacting with the SNARE complex and contributing to the precise control of vesicle fusion and neurotransmitter release. However, its role in the context of knee osteoarthritis (KOA) remains largely unknown. In order to verify the regulatory effect of CPLX2 on trafficking dynamics of DRG neurons and the influence of OPM on this process during KOA, we first observed changes in the expression level of VAMP2, SNAP25, and syntaxin1, key factors of membrane trafficking, with or without CPLX2 silencing in vivo. Immunofluorescence (Fig. 4a, b) showed the fluorescence intensity of VAMP2, SNAP25, and syntaxin1 in different groups of DRG tissues. The fluorescence intensity of VAMP2, SNAP25, and syntaxin1, were all significantly up-regulated in the KOA group compared with the Sham (*P* < *0.01*), MOPM intervention significantly reduced the up-regulation trend in the KOA group (*P* < *0.01*), and showed a stronger inhibitory effect after CPLX silencing (*P* < *0.01*). Besides, the differentially expressed gene (Fig. 4c) and protein (Fig. 4d, e) levels of the above factors also showed a consistent trend with the differentially expressed immunofluorescence intensity between groups (*P* < *0.05*).

### Membrane trafficking dynamics of DRG neurons in KOA is regulated by CPLX2 in vitro

**Fig. 5:**
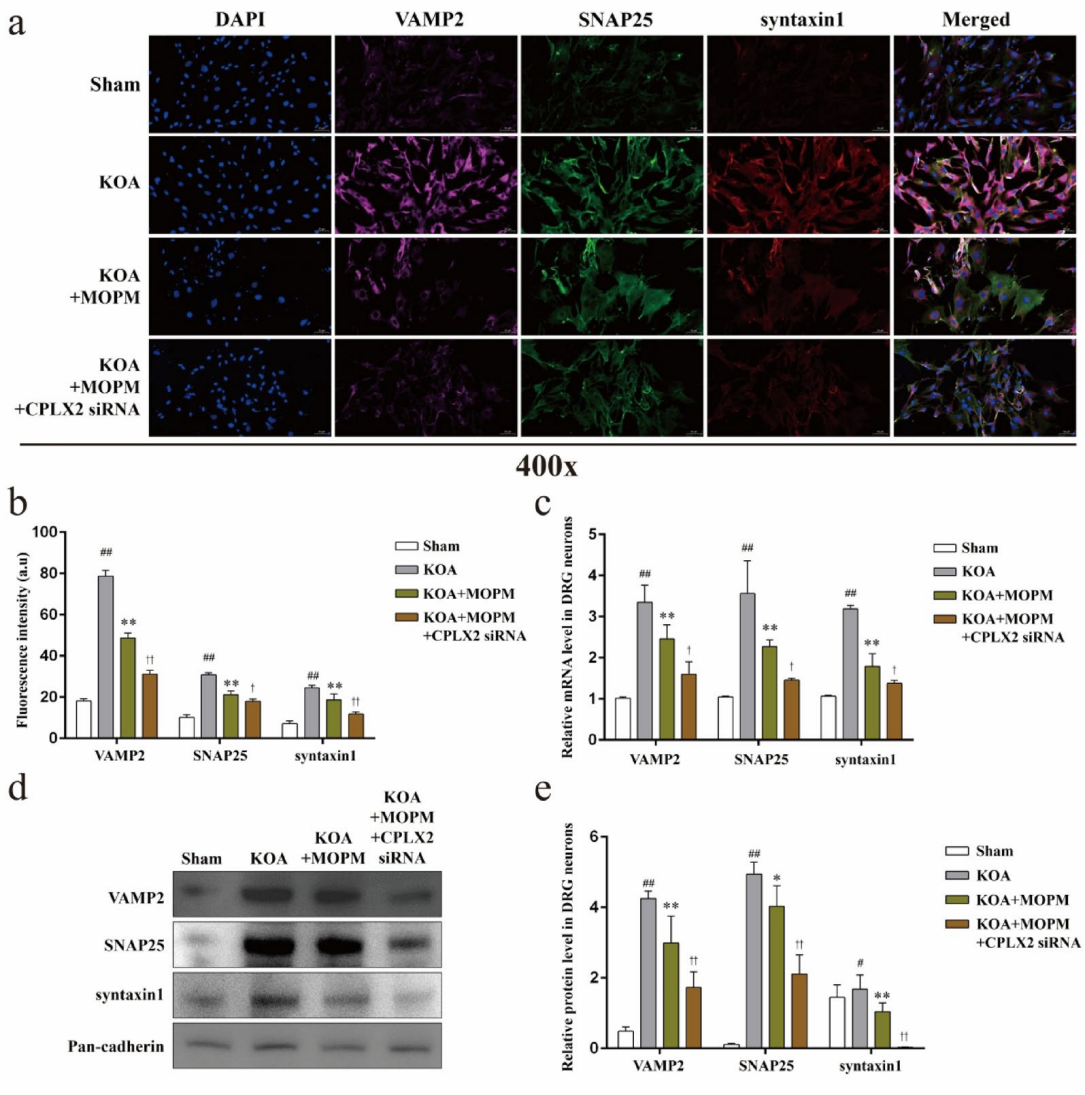
Membrane trafficking dynamics of DRG neurons in KOA is regulated by CPLX2 in vitro. *Notes*: **a**. Representative immunofluorescence of VAMP2 (purple), SNAP25 (green), and syntaxin1 (red) in DRG neurons. **b**. Fluorescence intensity statistics of VAMP2, SNAP25, and syntaxin1 in each group, ^##^*P* < 0.01 vs. Sham, ^**^P < 0.01 vs. KOA, ^†^*P* < 0.05, ^††^*P* < 0.01 vs. KOA + MOPM. **c**. Relative gene level of VAMP2, SNAP25, and syntaxin1 in DRG neurons, ^##^*P* < 0.01 vs. Sham, ^**^P < 0.01 vs. KOA, ^††^*P* < 0.05 vs. KOA + MOPM. **d**. Representative protein bands of VAMP2, SNAP25, and syntaxin1 in DRG neurons. **f**. Relative protein level of VAMP2, SNAP25, and syntaxin1 in DRG neurons, ^#^*P* < 0.05, ^##^*P* < 0.01 vs. Sham, ^*^P < 0.05, ^**^P < 0.01 vs. KOA, ^††^*P* < 0.01 vs. KOA + MOPM

In addition, we used the co-cultured OPM neurons and LPS-treated FLS to simulate the in vitro model of OPM intervention on KOA. In consistent with the results in vivo, the fluorescence intensity of VAMP2, SNAP25, and syntaxin1 in DRG neurons (Fig. 5a, b) were all significantly up-regulated in the KOA group compared with the Sham (*P* < *0.01*), MOPM intervention significantly reduced the up-regulation trend (*P* < *0.01*), and showed a stronger inhibitory effect after CPLX silencing (*P* < *0.05*). The differences in mRNA expression levels (Fig. 5c) of VAMP2, SNAP25, and syntaxin1 in neurons of all groups were consistent with the differences in fluorescence intensity (*P* < *0.05*). We also extracted DRG neuron membrane proteins with specific extraction kit, and WB results showed (Fig. 5d, e) an upregulated protein expression levels of the above factors in KOA neurons compared with the Sham (*P* < *0.01*), MOPM intervention downregulated these protein expressions compared with the KOA (*P* < *0.01*), while KOA + MOPM + CPLX2 siRNA further down-regulated the protein expression levels of these factors compared with KOA + MOPM group (*P* < *0.01*).

### Inhibition of CPLX2 enhanced the intervention of OPM on pain sensitivity and synovial inflammation in KOA in vivo

**Fig. 6:**
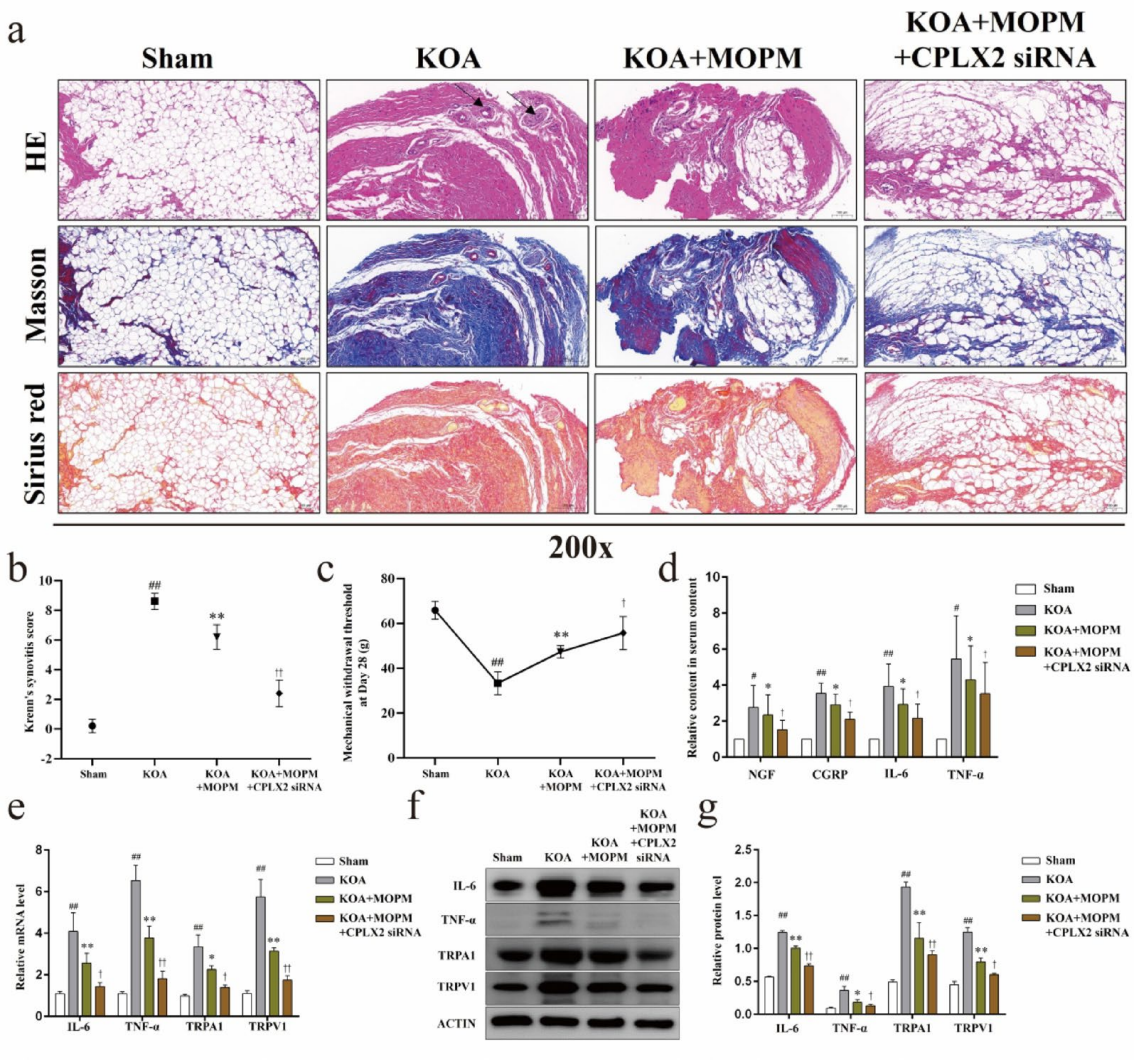
Inhibition of CPLX2 enhanced the intervention of OPM on pain sensitivity and synovial inflammation in KOA in vivo. *Notes*: **a**. Representative HE, Masson and Sirius red staining of rats’ synovial tissue in each group, the black arrows indicate infiltrating inflammatory cells. **d**. Krenn’s synovitis score of HE staining. **c**. MWT values of rats at Day28, ^##^*P* < 0.01 vs. Sham, ^**^P < 0.01 vs. KOA, ^†^*P* < 0.05, vs. KOA + MOPM. **d**. The serum content of NGF, CGRP, IL-6, and TNF-α, ^#^*P* < 0.05, ^##^*P* < 0.01 vs. Sham, ^*^P < 0.05, vs. KOA group, ^†^*P* < 0.05, vs. KOA + MOPM. **e**. Relative gene expression of IL-6, TNF-α, TRPA1 and TRPV1 in synovial tissues, ^##^*P* < 0.01 vs. Sham, ^*^P < 0.05, ^**^P < 0.01 vs. KOA, ^†^*P* < 0.05, ^††^*P* < 0.01, vs. KOA + MOPM. **f**. Representative protein bands of IL-6, TNF-α, TRPA1 and TRPV1 in synovial tissues. **g**. Relative protein level of IL-6, TNF-α, TRPA1 and TRPV1, ^##^*P* < 0.01 vs. Sham, ^*^P < 0.05, ^**^P < 0.01 vs. KOA, ^†^*P* < 0.05, ^††^*P* < 0.01, vs. KOA + MOPM

Further, we observed the in vivo effects of inhibited CPLX2 on pain sensitivity and synovial inflammation in KOA, and whether the intervention of OPM is dependent on this pathway. The synovial tissue of rats in KOA + MOPM + CPLX2 siRNA group showed an improved pathological staining results (Fig. 6a), as less inflammatory cell infiltration, ordered lining layer cells, and less collagen deposition, compared with the KOA + MOPM group. Consistently, Krenn’s synovitis score of synovial tissue HE staining (Fig. 6b) in KOA + MOPM group were higher than KOA + MOPM + CPLX2 siRNA group (*P* < *0.01*). Besides, MWT (Fig. 6c) in KOA + MOPM + CPLX2 siRNA group were significantly higher than KOA + MOPM group at Day28 (*P* < *0.05*), and the serum content of NGF, CGRP, IL-6, and TNF-α (Fig. 6d) were all downregulated (*P* < *0.05*). In addition, we detected the gene and protein expression of IL-6, TNF-α, TRPA1, and TRPV1 in synovial tissues of each group (Fig. 6e–g). CPLX2 siRNA significantly enhanced the inhibition effect of MOPM on the expression of these substances in KOA models (*P* < *0.05*).

### Inhibition of CPLX2 enhanced the intervention of OPM on pain sensitivity and synovial inflammation in KOA in vitro

**Fig. 7:**
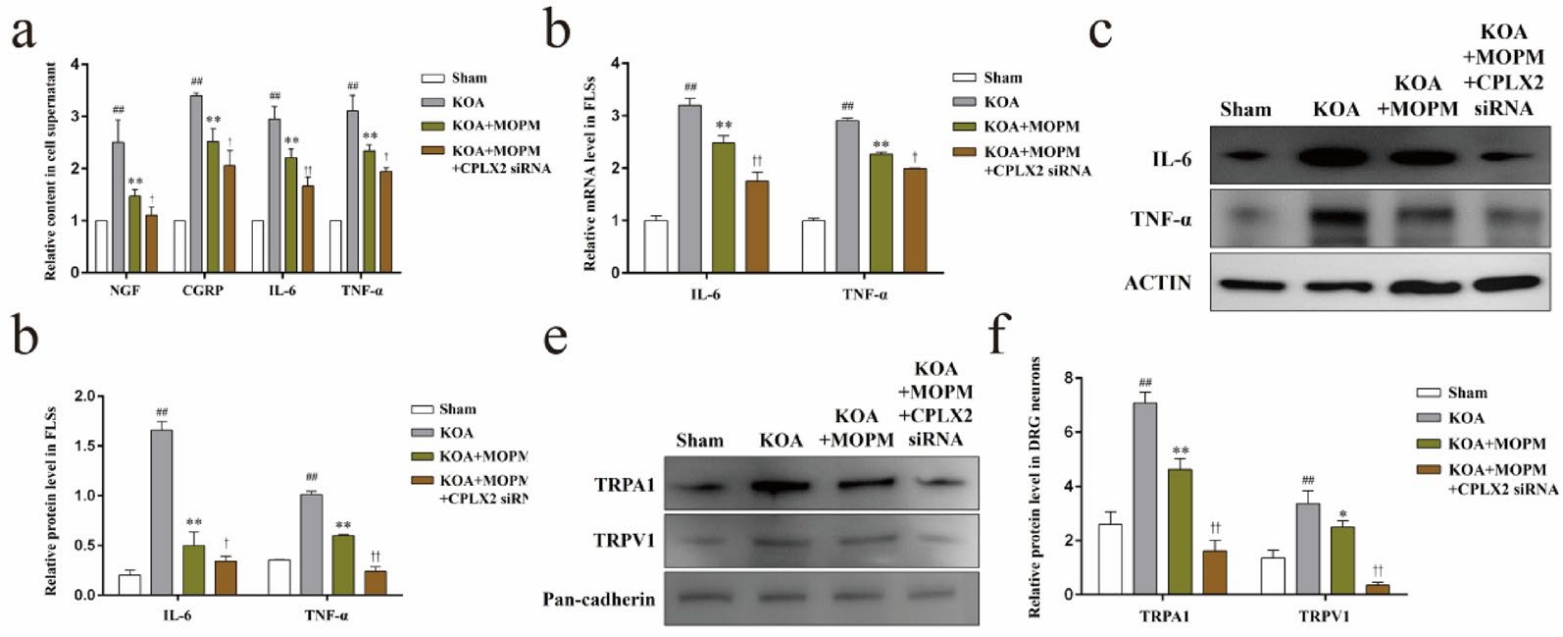
Inhibition of CPLX2 enhanced the intervention of OPM on pain sensitivity and synovial inflammation in KOA in vitro *Notes*: **a**. The contents of NGF, CGRP, IL-6, and TNF-α in the supernatant of cocultured cells, ^##^*P* < 0.01 vs. Sham, ^**^P < 0.01 vs. KOA, ^†^*P* < 0.05, ^††^*P* < 0.01, vs. KOA + MOPM. **b**. Relative gene level of IL-6 and TNF-α in FLSs, ^##^*P* < 0.01 vs. Sham, ^**^P < 0.01 vs. KOA, ^†^*P* < 0.05, ^††^*P* < 0.01, vs. KOA + MOPM. **c**. Representative protein bands of IL-6 and TNF-α in FLSs. **d**. Relative protein level of IL-6 and TNF-α in FLSs, ^##^*P* < 0.01 vs. Sham, ^**^P < 0.01 vs. KOA, ^†^*P* < 0.05, ^††^*P* < 0.01, vs. KOA + MOPM. **e**. Representative protein bands of TRPA1, TRPV1 in the cell membrane of DRG neurons. **f**. Relative protein expression of TRPA1, TRPV1 in the cell membrane of DRG neurons, ^##^*P* < 0.01 vs. Sham, ^*^P < 0.05, ^**^P < 0.01 vs. KOA, ^††^*P* < 0.01, vs. KOA + MOPM

In consistent with the above in vivo results, contents of NGF, CGRP, IL-6, and TNF-α in the supernatant of cocultured cells (Fig. 7a) were significantly higher in KOA + MOPM group than KOA + MOPM + CPLX2 siRNA group (*P* < *0.05*). Both gene and protein expression of IL-6, TNF-α in FLSs (Fig. 7b–d), and protein expression of TRPA1, TRPV1 in the cell membrane of DRG neurons (Fig. 7e, f) were significantly down-regulated in KOA + MOPM + CPLX2 siRNA group compared with KOA + MOPM group (*P* < *0.05*).

## Discussion

TCSOM is a subject of continuous inheritance and innovation, rooted in the basic theory of traditional Chinese medicine. This method was first recorded in the “Yellow Emperor’s Inner Classic” and among the common method of chiropractic treatment, OPM ranks first [31, 32]. However, current studies have not been able to explain the molecular biological mechanism of its therapeutic effect on musculoskeletal muscle system diseases. In this study, we first tried to construct the in vivo and in vitro systems to simulate the OPM operation process. We designed OPM device for small animals, which is composed of sleeve, spring, connecting rod and rubber cap. This enabled us to successfully perform OPM intervention on KOA rats, and we also realized OPM intervention on KOA in vitro by FX-6000 T system and co-cultured DGR neurons with FLSs cells. We confirmed that moderate OPM can significantly upregulated MWT and improve synovial inflammation and fibrosis in KOA rat, reduce the content of pain mediators NGF, CGRP and proinflammatory factors IL-6, TNF-α, and down-regulate the expression of IL-6, TNF-α, TRPA1, TRPV1. It should be emphasized that low-intensity OPM seems to be ineffective in the intervention of KOA, while high-intensity OPM, although effective, is actually beyond the clinical use intensity.

Subsequently, RNA sequencing of DRG tissues revealed that MOPM reversed KOA-induced upregulation of CPLX2, a key modulator of SNARE complex-mediated cellular membrane trafficking. CPLX2 is an important member of the complexin family, interact with the SNARE complex, and contribute to the precise control of vesicle fusion and neurotransmitter release [33, 34]. In this study, we found the regulatory effect of CPLX2 on SNARE complex-mediated DRG membrane trafficking in KOA model, both gene and protein levels of VAMP2, SNAP25, and syntaxin1 were all significantly up-regulated in the KOA group compared with the Sham. MOPM intervention significantly reduced the up-regulation trend in the KOA group, and showed a stronger inhibitory effect after CPLX2 silencing. This indicates that DRG neurons in KOA have more active cell membrane trafficking, CPLX2 is the key factor, and MOPM can prevent DRG cell membrane trafficking in KOA by reducing the expression of CPLX2. Furthermore, although the present study did not provide direct evidence, previous studies have reported a correlation between the expression of CPLX2 and nuclear respiratory factor 1 [35]. The protective effect of activating the NRF signaling pathway in KOA has been well demonstrated in numerous studies [36, 37].

SNAP-25 is highlighted as a crucial component of the SNARE complex. This complex, formed with syntaxin-1 and VAMP2, is essential for membrane fusion during neurotransmitter release [38, 39]. Besides, researches indicate that SNAP-25 expression increases in the dorsal root ganglia (DRG) of melanoma mouse model, or chronic sciatic nerve pain rats’ model, during tumor-induced pain [40] or nerve injury- induced pain [41]. VAMP2 is another key component of the SNARE complex, and plays a major role in the transportation of TRPA1 and TRPV1 [42–44]. Experiments using lentiviral vectors showed TRPV1-DsRed and VAMP2-GFP showed co-localize intracellular distributions, and TNFα treatment resulted in more surface TRPV1 in cells expressing VAMP2-GFP [39]. Due to the high consistency of TRPV1 and TRPA1 in cytoplasmic to cell membrane trafficking, this study also confirmed that VAMP2 is primarily involved in the membrane insertion of TRPV1 and TRPA1 in sensory neurons [39].

In our study, we affected DRG neuronal cell membrane trafficking in KOA by intervening with CPLX2, and we observed that when the membrane trafficking process was inhibited, both gene and protein levels of inflammatory factors IL-6, TNF-α, nociceptor TRPA1, TRPV1 were significantly decreased in KOA model, as well as the content of pain mediators NGF and CGRP. MOPM has a clear blocking effect on KOA synovial inflammation and nociceptive hypersensitivity, which is realized through CPLX2-mediated membrane trafficking. When CPLX2 is blocked, the intervention effect of MOPM on KOA will be further enhanced.

Nevertheless, our study has certain limitations. First, we did not assess histopathological changes in cartilage before and after OPM intervention in KOA rats, which prevented us from evaluating KOA progression from the perspective of whole-joint pathology. Moreover, the mechanisms underlying nociceptive hypersensitivity in KOA are multifactorial. In addition to synovial inflammation and nociceptor innervation of the synovium, nerve sprouting in the subchondral bone and its invasion into cartilage are also important contributors to KOA-associated nociceptive hypersensitivity. Second, the DRG is likely subjected predominantly to compressive rather than tensile forces in vivo, which limits the relevance of our in vitro tensile loading system. Isolating DRG neurons immediately after in vivo interventions at defined time points could address this issue, and we consider this a promising direction for future research. Third, the device we used to simulate OPM was relatively rudimentary and lacked standardized quality control measures to ensure its reliability. Future studies should focus on optimizing and validating this apparatus. Finally, we acknowledge that our immunofluorescence images provide only a preliminary visualization of the localization of VAMP2, SNAP25, and syntaxin1. Further studies are warranted to investigate their precise subcellular localization, clarify the spatial relationships among these proteins, and explore their involvement in the trafficking processes of TRP proteins.

## Conclusions

In conclusion, this study demonstrates that OPM alleviates KOA-associated nociceptive hypersensitivity and synovitis by targeting CPLX2-mediated trafficking dynamics of transient receptor potentials in DRG neurons. These results provide a mechanistic foundation for OPM’s clinical use and identify CPLX2 as a promising target for KOA management. Future studies should explore OPM’s long-term efficacy in human trials and its interplay with other membrane trafficking regulators.

## Supplementary Information

Below is the link to the electronic supplementary material.

Supplementary Material 1

## Data Availability

The data used to support the findings of this study are available from the corresponding author upon request.

## Abbreviations

KOA: Knee osteoarthritis
TRPs: Transient receptor potentials
IL-6: Interleukin-6
TNF-α: Tumor necrosis factor-α
SNARE: Soluble N-ethylmaleimide-sensitive factor attachment protein receptor
VAMP2: Vesicle-associated membrane protein 2
SNAP-25: Synaptosomal-associated protein of 25 kDa
CGRP: Calcitonin gene related peptide
NGF: Nerve growth factor
DRG: Dorsal root ganglion
TCSOM: Traditional Chinese spinal orthopedic manipulation
OPM: Osteopathic pressing manipulation
FLSs: Fibroblast-like synoviocytes
