# Supplementary material for: Mechanical stimulation simulating osteopathic pressing manipulation inhibits nociceptive hypersensitivity and synovial inflammation in knee osteoarthritis rats by modulating trafficking dynamics of transient receptor potentials via complexin2
Source: J Orthop Surg Res. 2025 Nov 5;20:962. doi: 10.1186/s13018-025-06414-7 (PMC12590787; doi:10.1186/s13018-025-06414-7)

original western blot for three repeats-Figure1

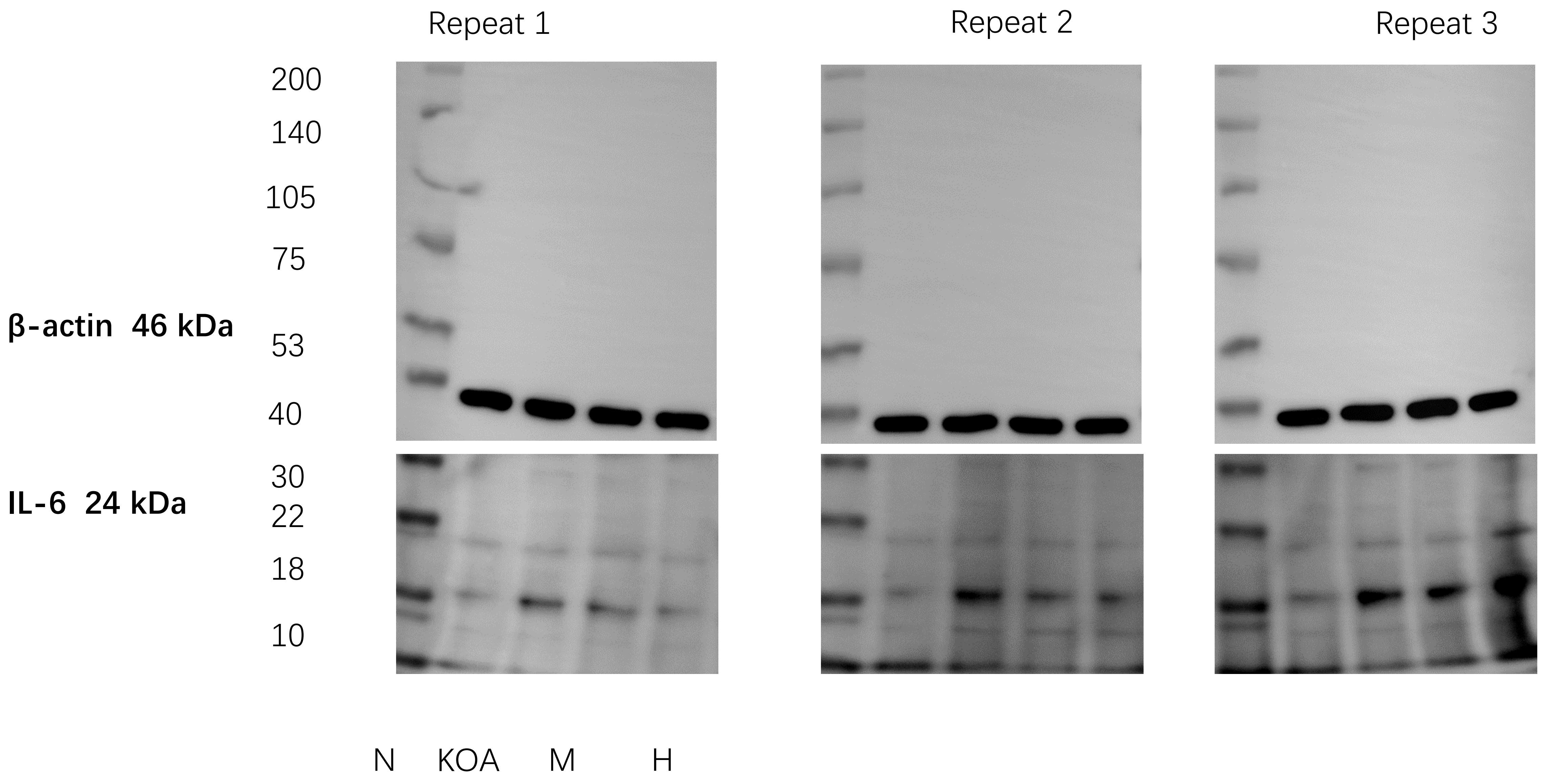

original western blot for three repeats-Figure1

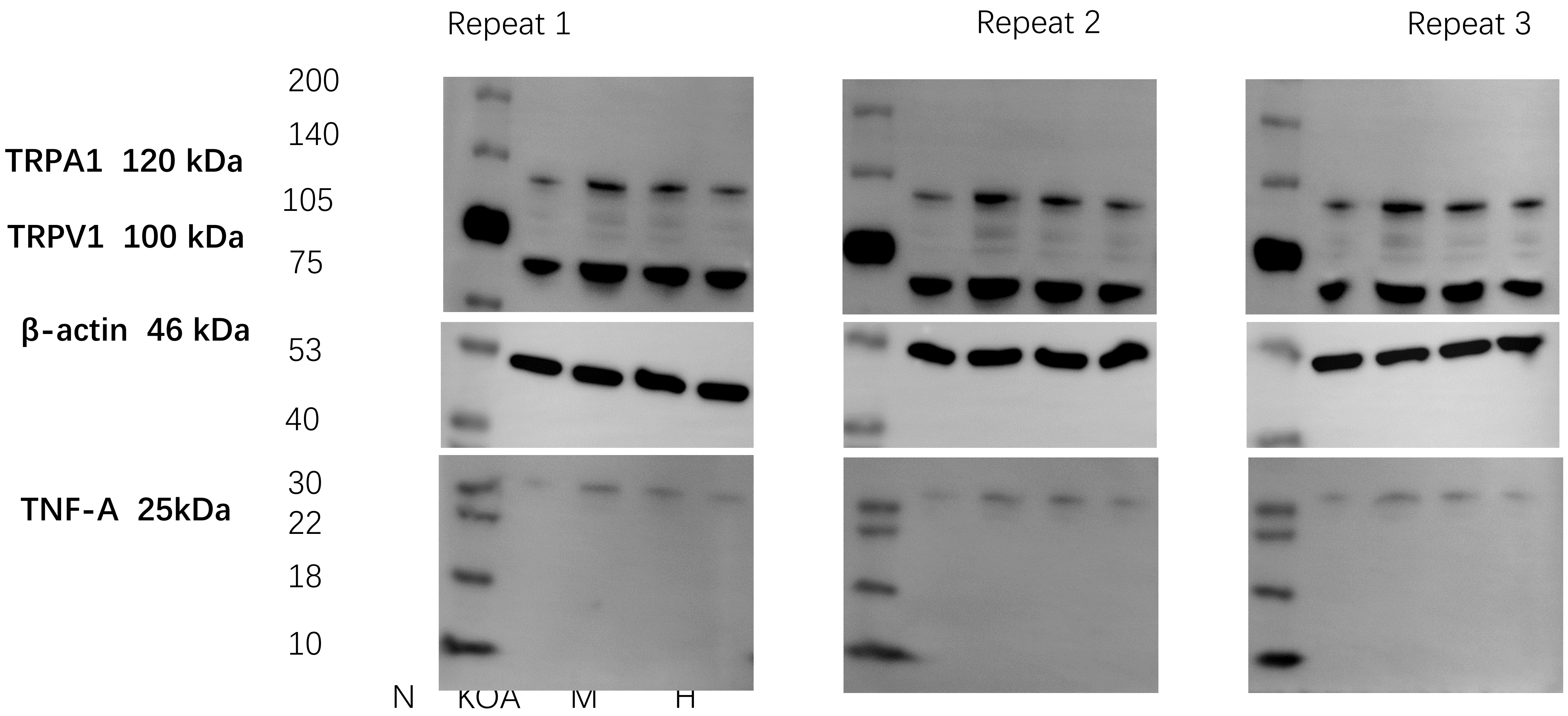

original western blot for three repeats-Figure2

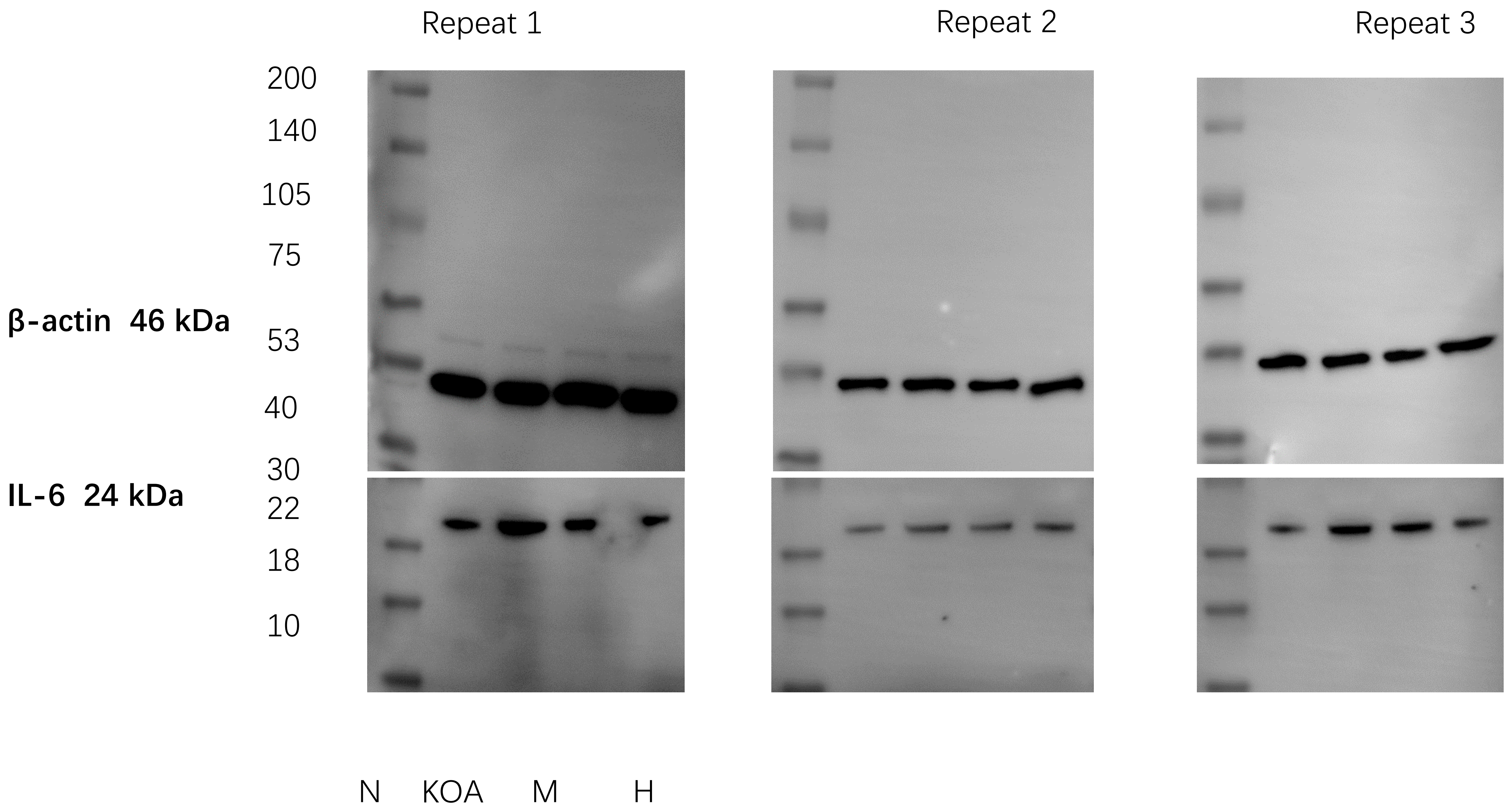

original western blot for three repeats-Figure2

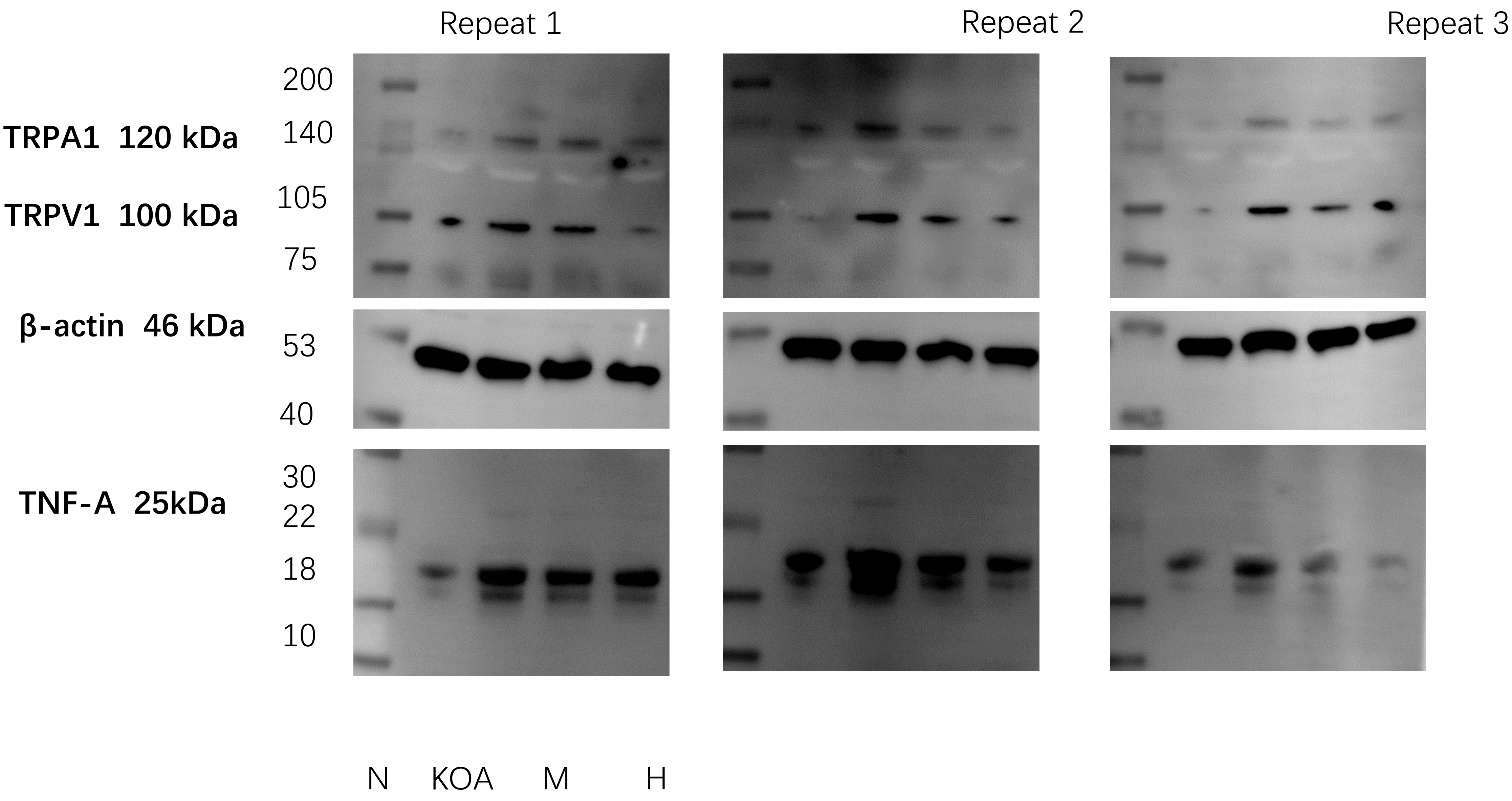

original western blot for three repeats-Figure4

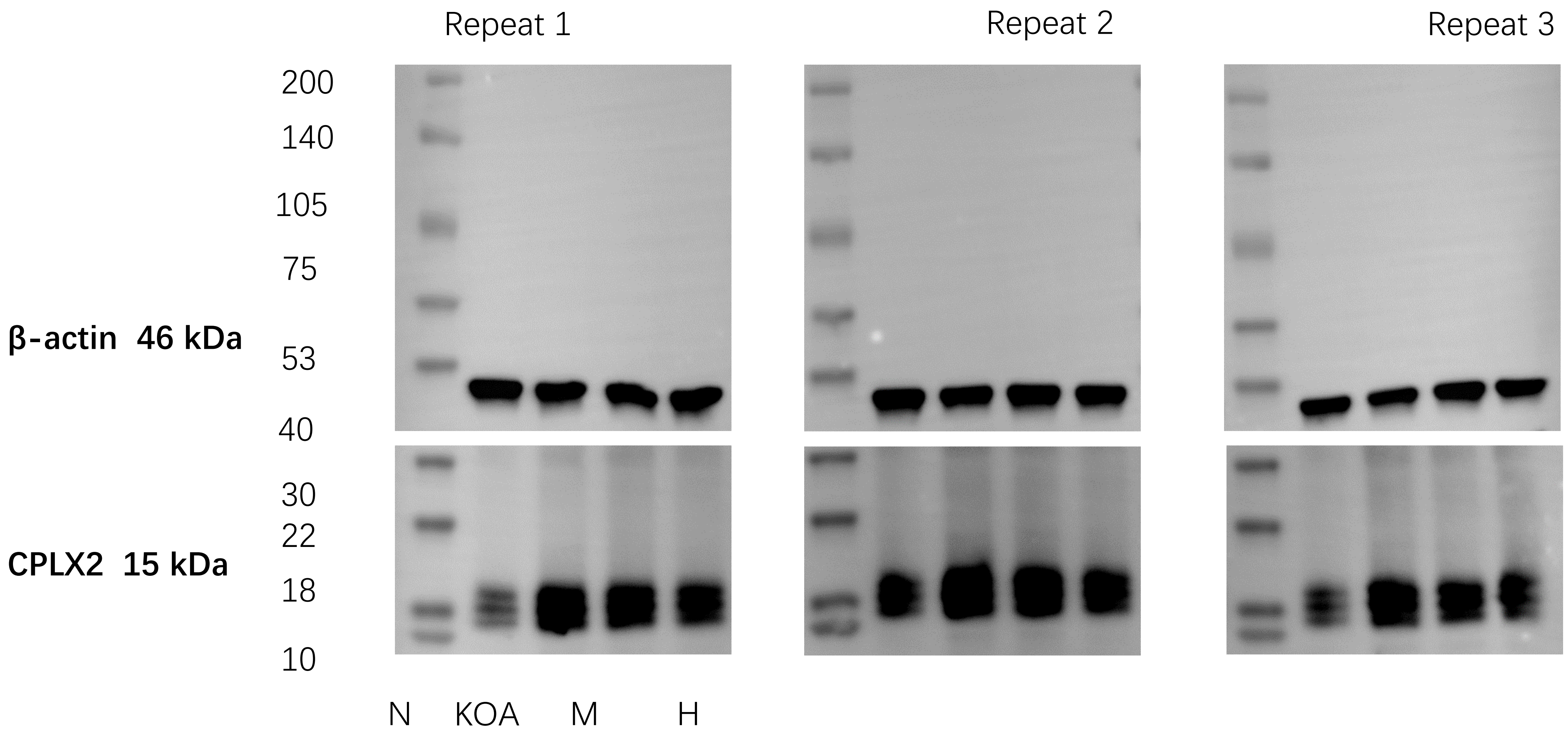

original western blot for three repeats-Figure4

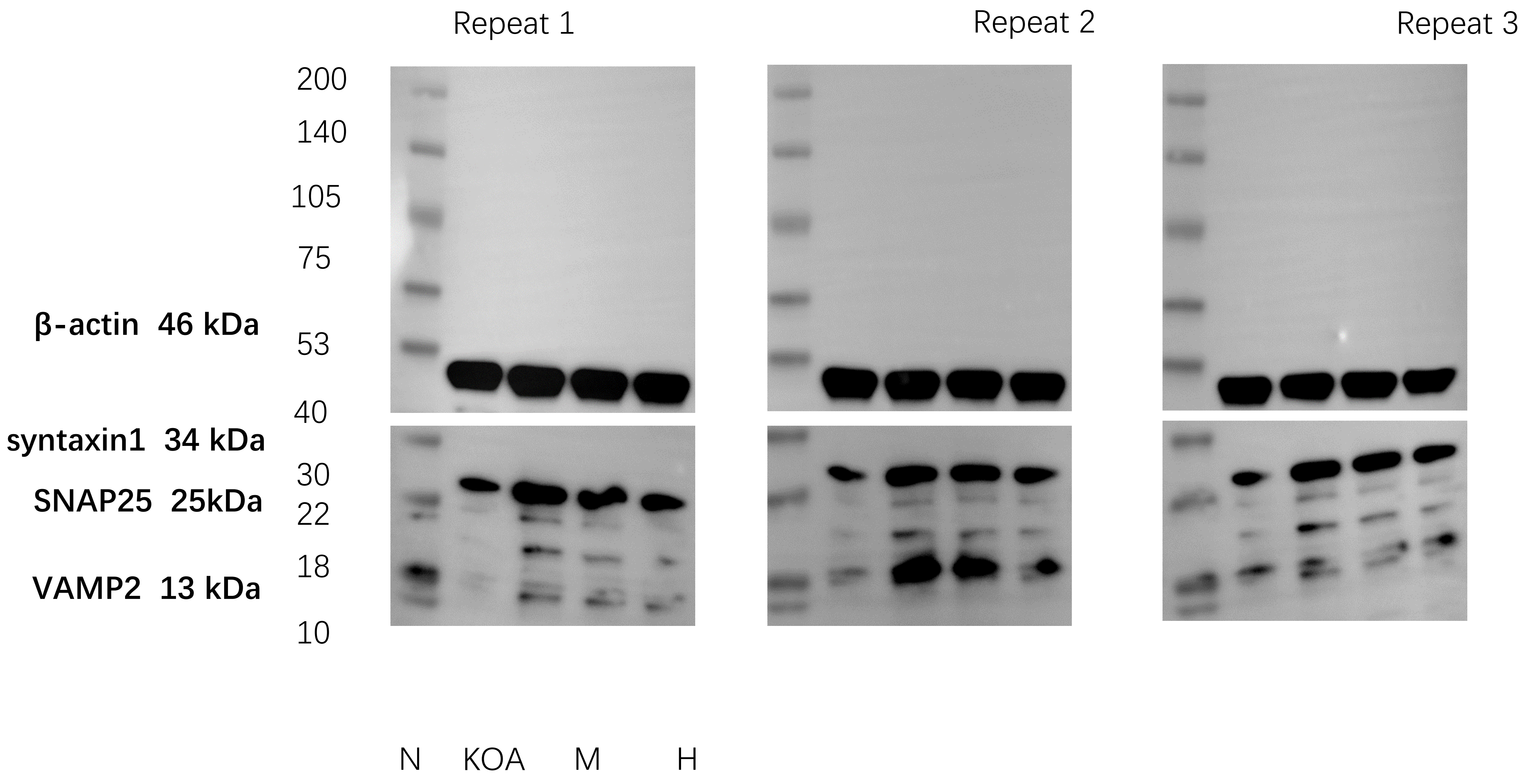

original western blot for three repeats-Figure5

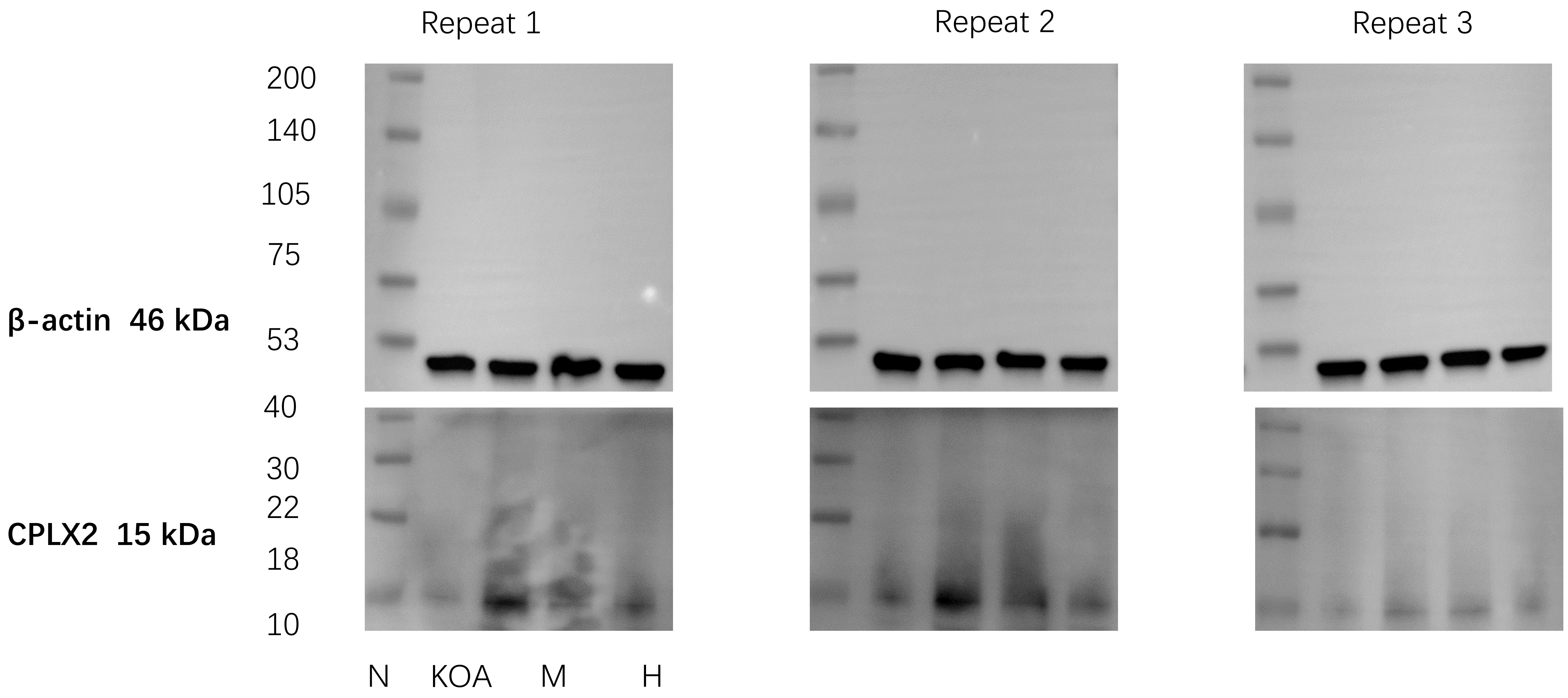

original western blot for three repeats-Figure5

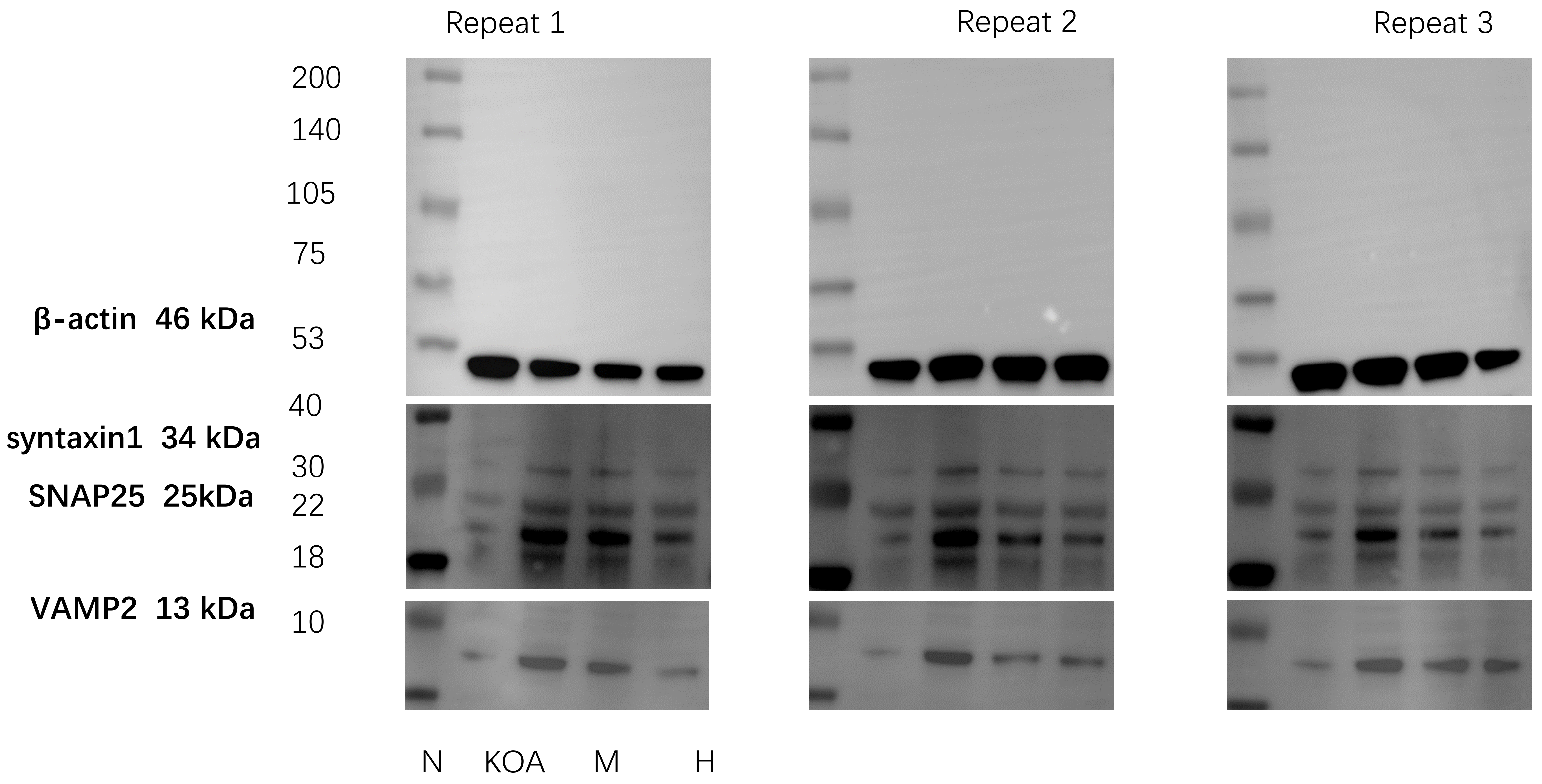

original western blot for three repeats-Figure6

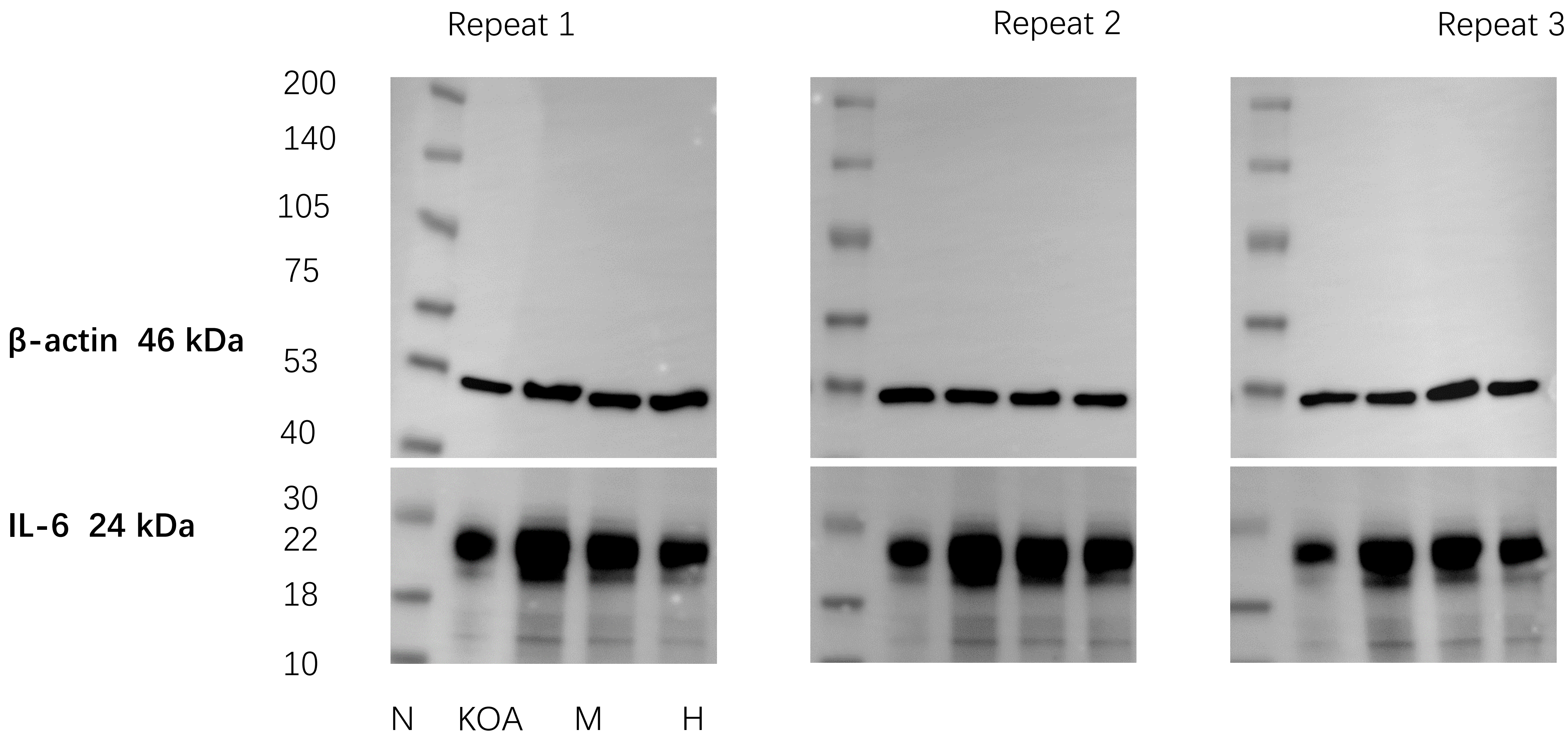

original western blot for three repeats-Figure6

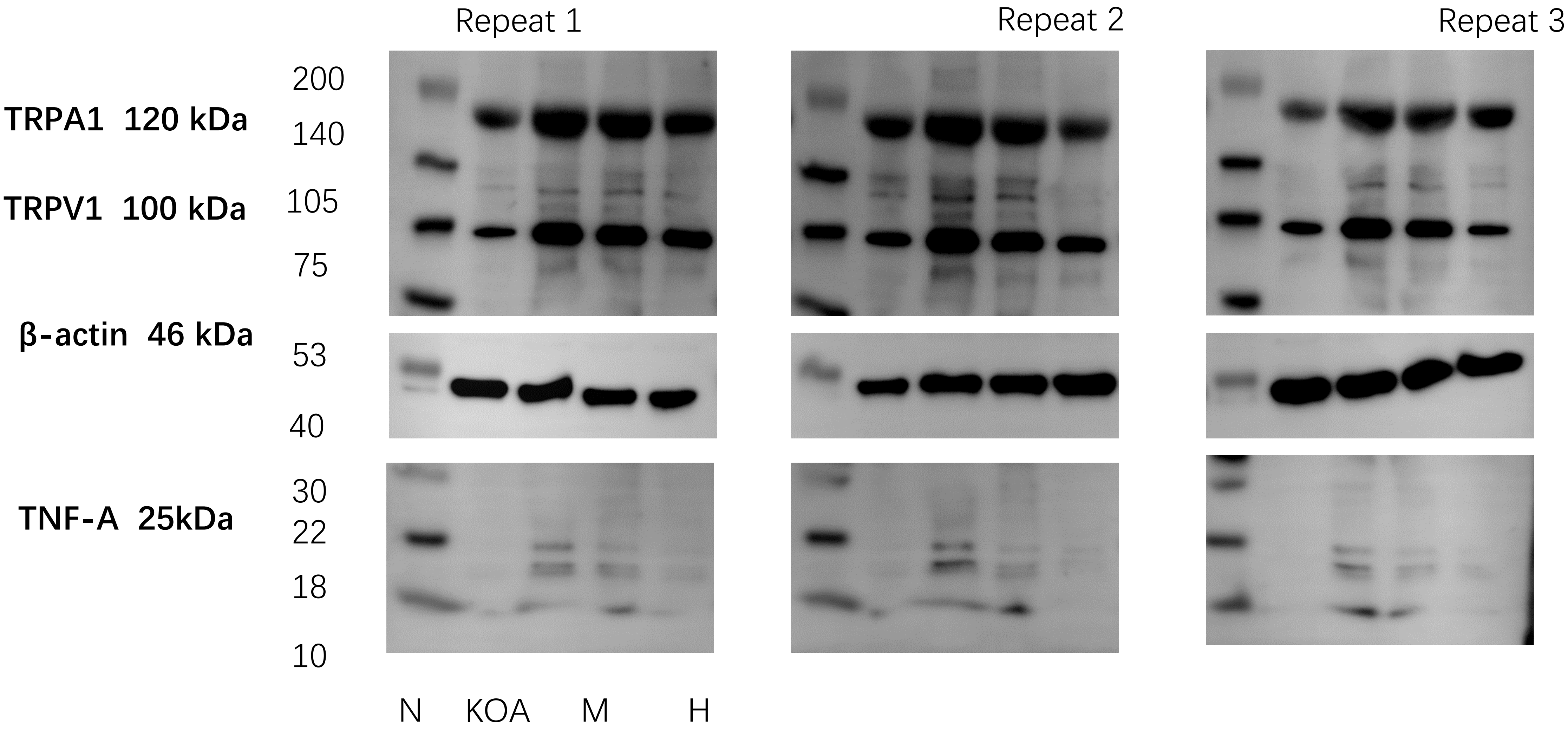

original western blot for three repeats-Figure7

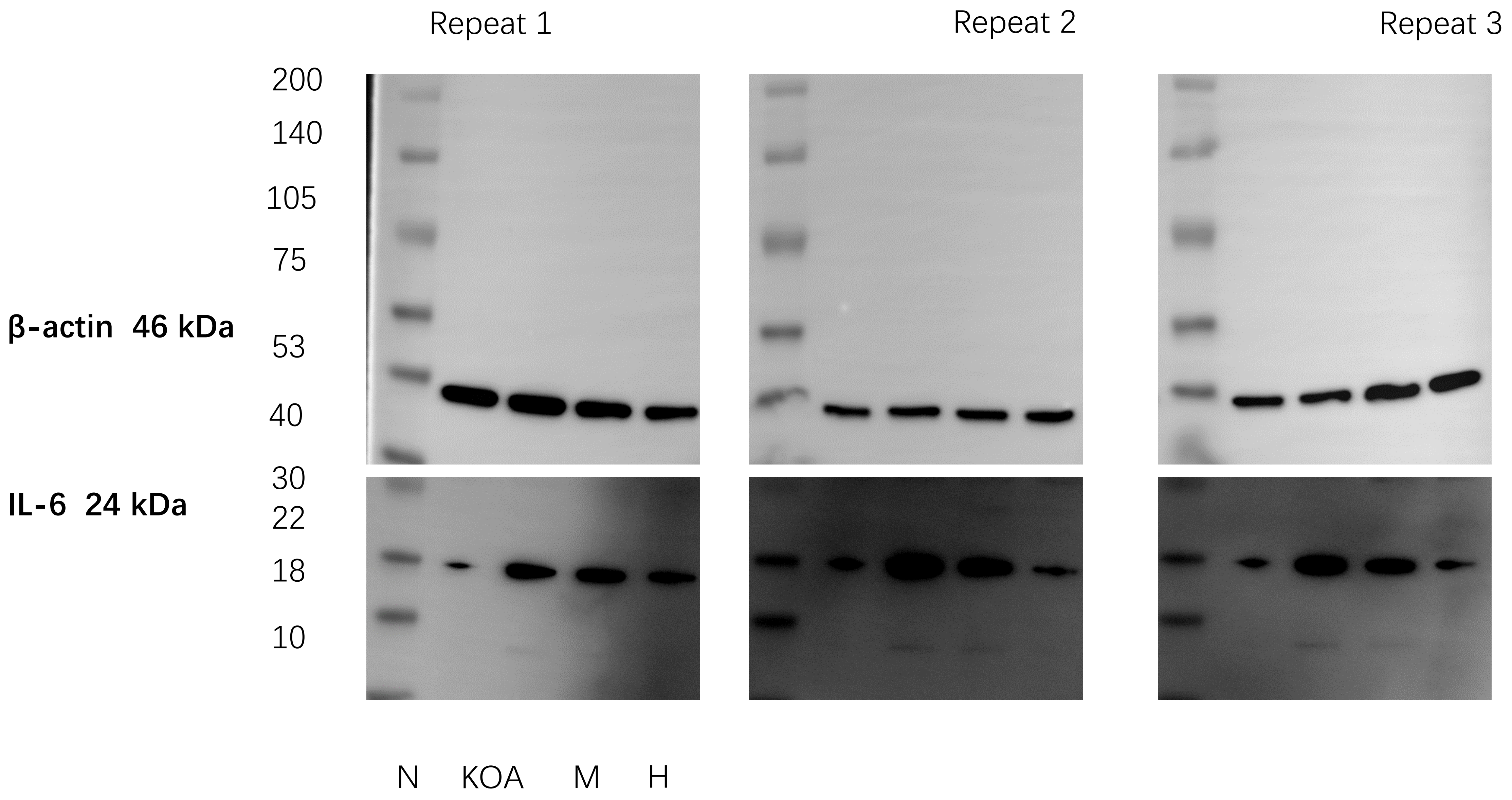

original western blot for three repeats-Figure7

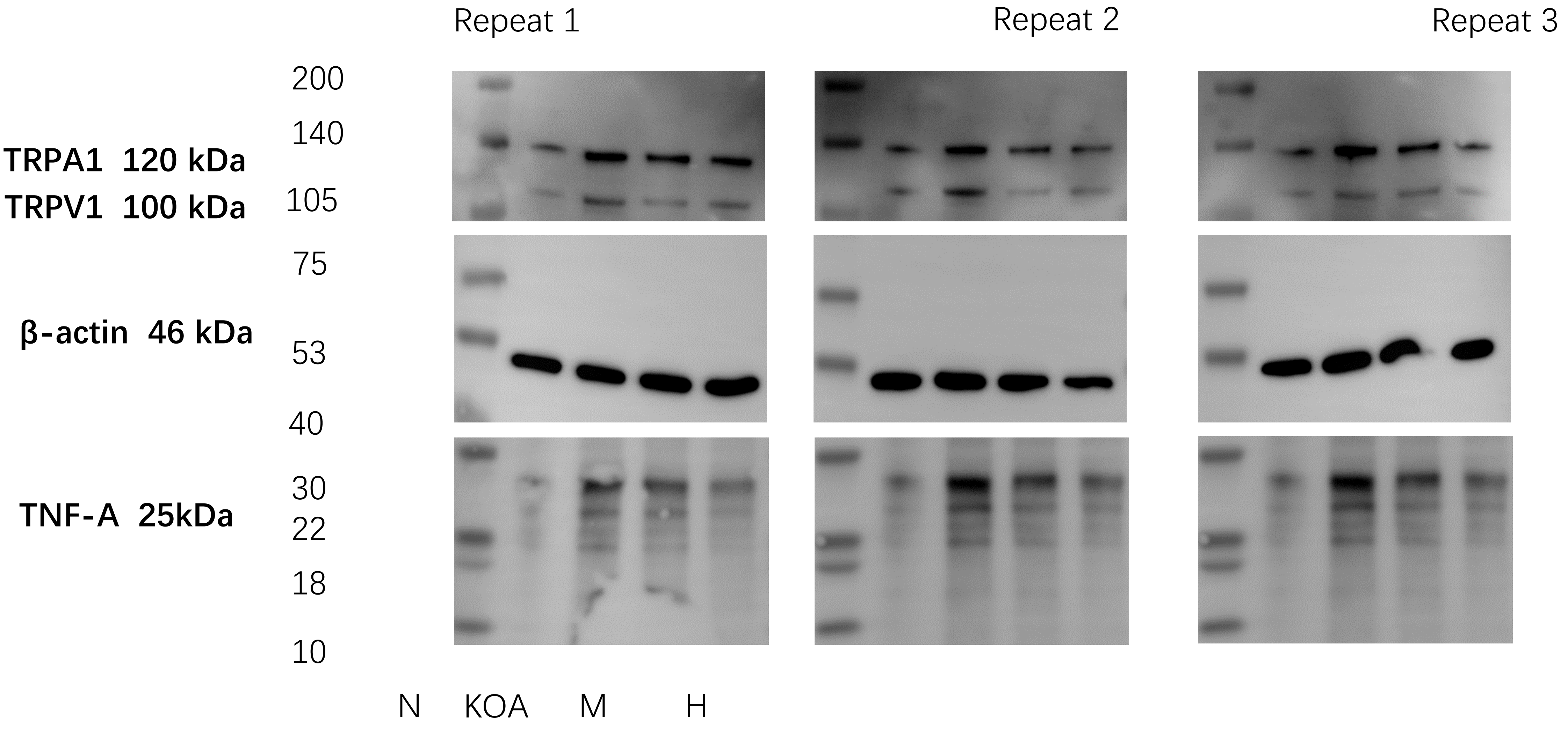

Supplement: Supplementary file 1 — Supplementary Material 1 [file 13018_2025_6414_MOESM1_ESM.pdf]
